# TopBP1 orchestrates PU.1–IRF8 transcriptional programming of dendritic cell differentiation and Flt3L-driven tumor immunity

**DOI:** 10.1038/s12276-026-01715-1

**Published:** 2026-05-08

**Authors:** Min-Suk Cha, Myeong-Ho Kang, Jinjoo Lee, JungHyub Hong, Yu Sun Jeong, Yoe-Sik Bae, Ho Lee, Seok-Hee Park, Yong-Soo Bae

**Affiliations:** 1 Department of Biological Sciences, Sungkyunkwan University, Suwon, Republic of Korea; 2 Center for Immune Research on Non-Lymphoid Organs, Sungkyunkwan University, Suwon, Republic of Korea; 3 Department of Health Sciences and Technology, Samsung Advanced Institute for Health Sciences and Technology, Sungkyunkwan University, Seoul, Republic of Korea; 4 Division of Convergence Technology, Research Institute, National Cancer Center, Goyang, Republic of Korea

**Keywords:** Conventional dendritic cells, Cancer immunotherapy

## Abstract

DNA topoisomerase II-binding protein 1 (TopBP1) plays a critical role in V(D)J recombination and DNA damage repair during B and T cell development. However, its role in the development of conventional dendritic cells (cDCs) remains unexplored. Mice with DC-specific depletion of TopBP1 (TopBP1^cKO^) exhibited accelerated tumor progression due to impaired anti-tumor immunity, which was characterized by cDC deficiency and pre-DC accumulation. The cDC deficiency observed in TopBP1^cKO^ mice was not attributable to cell death resulting from accumulated DNA damage during DC development. Notably, Flt3 ligand (Flt3L)-mediated tumor immunotherapy was ineffective in TopBP1^cKO^ tumor-bearing mice. Here we demonstrate that TopBP1 is required not only for the steady-state differentiation of total cDCs, including both cDC1 and cDC2, but also for the terminal differentiation of XCR1^−^CD24⁺ emergency progenitors (CD11c⁺cKit⁺) into XCR1⁺CD24⁺ cDC1s in response to Flt3L. Furthermore, TopBP1 was found to be essential for the function of the PU.1–IRF8 heterodimeric transcription factor complex, which is critical for cDC lineage specification. TopBP1 directly binds to this complex and facilitates the transcription of downstream target genes required for cDC development. These findings establish TopBP1 as a pivotal regulator of both steady-state and Flt3L-driven emergency cDC differentiation, particularly in guiding emergency progenitors into functional cDC1s. Our study highlights the previously unrecognized role of TopBP1 as a co-regulator of lineage-defining transcription factors and as a determinant of Flt3L-mediated anti-tumor efficacy.

## TopBP1 essential for dendritic cell development and immunity

This study explores the role of topoisomerase II binding protein 1 (TopBP1) in dendritic cell (DC) development, particularly conventional DCs (cDCs), which are crucial for immune responses. The authors reveal that TopBP1 directly interacts with the PU.1–IRF8 heterodimeric complex, a novel regulatory axis essential for cDC differentiation. Using TopBP1 conditional knockout (TopBP1^cKO^) mice, they demonstrate impaired cDC development, leading to rapid tumor growth and ineffective Flt3L-based tumor immunotherapy. The study employed various methods, including flow cytometry, co-immunoprecipitation and RNA sequencing, to elucidate TopBP1’s role in cDC differentiation. Results show that TopBP1 is critical for the transition of XCR1^−^CD24^+^ emergency progenitors into XCR1^+^CD24^+^cDC1s, highlighting its importance in immune regulation. These findings suggest potential therapeutic targets for enhancing DC-mediated immunity and point to future research on TopBP1’s interactions with transcription factors in DC biology.

This summary was initially drafted using artificial intelligence, then revised and fact-checked by the author.

## Introduction

Most cells undergo multiple stages of proliferation and differentiation to reach their final cell types. Substantial DNA damage occurs during these processes, which must be properly repaired to prevent cell death. Topoisomerase II binding protein 1 (TopBP1) is one of the key regulator of DNA damage repair^1–4^ and is also involved in cell cycle checkpoint regulation^5–7^, DNA replication initiation^8,9^, transcriptional regulation^10,11^ and cell survival^12,13^. TopBP1 is induced during the G1/S transition, where it interacts with Treslin and CDC45 to initiate DNA replication^9,14^. In response to DNA damage, TopBP1 interacts with ATR^15^ and ATM^1,16^, facilitating DNA repair and enabling cell cycle progression. Akt phosphorylates TopBP1 at Ser1159, inducing its oligomerization^10,11^. Oligomerized TopBP1 inhibits its association with chromatin and ATR, reducing its function in activation of ATR. Instead, oligomerized TopBP1 is associated with E2F1 and inhibits E2F1-dependent cell apoptosis^11^. In addition, TopBP1 is crucial for cell survival^12^, and its complete knockout causes embryonic lethality and neurogenesis defects^17,18^. In immunological studies, TopBP1 is essential for V(D)J recombination and development of B, T and iNKT cells through double-strand break (DSB) damage repair^19^.

Dendritic cells (DCs) are professional antigen-presenting cells that bridge innate and adaptive immunity by presenting antigens to specific T cells. DCs can be broadly categorized into conventional DCs (cDC) and plasmacytoid DCs (pDC)^20^. pDCs play an important role in anti-viral immunity by secreting large amounts of type I interferon, whereas cDCs initiate and induce adaptive immune responses against foreign invaders and cancers. Under steady-state conditions, hematopoietic stem cells give rise to common DC progenitors through several intermediate progenitor stages in the bone marrow (BM). These common DC progenitors possess the capacity to differentiate into pDCs and pre-CDCs (pre-DCs; Lin^−^CD135⁺MHCII^−^CD11c⁺CD115^−^CD117^−/int^) within the BM. Pre-DCs migrate through the blood to the respective organs where they further differentiate into cDC subsets (XCR1^+^cDC1, CD11b^+^cDC2) depending on the microenvironment^21–24^. The Flt3 ligand (Flt3L), a growth factor necessary for pre-DCs and cDC1 differentiation, promotes cDC1-mediated anti-tumor immunity and is widely used in cancer immunotherapy^25^. It has been reported that Flt3L increases the level of emergency progenitors (EPs; CD11c^+^cKit^+^ population), which are normally present in very small numbers in BM and that the EPs expanded by Flt3L ultimately differentiate into IRF8-dependent cDC1s^26^. Meanwhile, GM-CSF promotes cDC2 and monocyte-derived DC differentiation.

Many transcription factors (TFs) have been identified as essential for DC subset specification. For instance, E2-2 is critical for the differentiation and maintenance of pDCs^27–29^; IRF8^30,31^, ID2^32,33^, Batf3^34^ and Nfil3^32,33,35^ are indispensable for cDC1 development, whereas IRF4^36,37^, Notch2^38^ and Klf4^39,40^ are important for cDC2 differentiation^39,41^. However, despite this extensive characterization of subset-specific TFs, the transcriptional regulation of total cDC differentiation, including both cDC1 and cDC2, remains poorly understood. So far, the Ets-family protein PU.1, encoded by Spi1, is the only well-established TF essential for the overall differentiation and function of total cDCs including cDC1 and cDC2^42,43^. PU.1 is highly expressed in cDCs, where it maintains identity and promotes formation, particularly of cDC1s, by inducing the transcriptional regulator DC-SCRIPT but inhibits the development of pDCs^43^. PU.1 is also required for differentiation of other immune cells^44,45^.

TopBP1 has not previously been reported to be involved in DC biology. To explore a potential connection between TopBP1 and DC-specific TFs, we examined known TopBP1-interacting partners and found that TopBP1 interacts with Miz-1 (Myc-associated zinc-finger protein). Miz-1 is a TF known to interact with MYC and regulate the transcription of cell cycle inhibitory genes (for example, p15^INK4b^). Miz-1 also stabilizes TopBP1 by forming a complex with HectH9, which inhibits TopBP1 ubiquitination and protects it from proteasomal degradation, thereby maintaining its role in cell cycle checkpoint control during DNA damage responses^46^. Also, Miz-1 has been shown to interact with IRF8 to activate the Nramp1 promoter, thereby contributing to innate resistance against intraphagosomal pathogens^47^. This promoter activation is further enhanced by PU.1. These findings were identified through yeast two-hybrid screening using macrophage cell line^47^. However, no prior studies have demonstrated whether TopBP1 directly associates with DC- related TFs. In this study, we provide in vivo evidence that TopBP1 directly interacts with PU.1–IRF8 heterodimeric complex in DCs, revealing a novel regulatory TopBP1–PU.1–IRF8 axis critical for cDC development.

This study demonstrates that (1) TopBP1 is highly expressed in DC precursors and cDCs, (2) TopBP1^cKO^ mice exhibited accelerated tumor growth and Flt3L tumor immunotherapy was ineffective in these mice, (3) TopBP1 is essential for the differentiation of pre-DC into cDCs under steady-state conditions, (4) TopBP1 is specifically required for the final differentiation of XCR1⁻CD24⁺ EPs into XCR1⁺CD24⁺ cDC1s and (5) TopBP1 directly interacts with PU.1–IRF8 heterodimeric complex to facilitate cDC development and Flt3L-mediated cDC1 differentiation.

## Materials and methods

### Mice

Topbp1^fl/fl^ mice were provided by Dr. Ho Lee of the National Cancer Center. CD11c-cre (B6.Cg-Tg(Itgae-cre)1-1Reiz/J) and OT-I (C57BL/6-Tg(TcraTcrb)1100Mjb/J) mice were purchased from Jackson Laboratory. Topbp1^fl/fl^ mice were crossed with CD11c-cre mice. Mice 7–10 weeks of age were used in these studies. All mice were bred and maintained under specific pathogen-free conditions at the Sungkyunkwan University Lab Animal Resource Center. All procedures for animal experiments were performed according to University Animal Care and Use guidelines.

### Cell lines and reagents

C57BL/6 mouse origin EL4, E.G7 (OVA-expressing EL4; lymphoma), B16F10 (melanoma), B16F10-Flt3L (Flt3L-expressing B16F10), B16F10-GM-CSF (GM-CSF-expressing B16F10) and human embryonic kidney 293 (HEK293) cells were purchased from American Type Culture Collection (ATCC). E.G7 was cultured in RPMI1640 (Gibco) with 10% FBS (Gibco) and 100 U/ml penicillin–streptomycin (Gibco), and B16F10 and HEK293 cells were cultured in DMEM (Gibco) with 10% FBS and 100 U/ml penicillin–streptomycin at 37 °C and 5% CO_2_. All cell lines were routinely tested for mycoplasma contamination by PCR.

### Cell isolation from tissues

Spleens and lymph nodes (LNs) were ground and passed through a 70-μm strainer. Livers, kidneys and lungs were collected after perfusion and treated with 0.1 mg/ml type IV collagenase (Sigma-Aldrich) for ~30–45 min at 37 °C. Collagenase-treated livers and lungs were passed through a 70-μm strainer and washed with phosphate-buffered saline (PBS). The cells isolated from spleens, LNs, livers, kidneys and lungs were treated with ACK buffer (Lonza) for erythrocyte lysis, and the cells were washed with PBS and analyzed by flow cytometry.

### Flow cytometry analysis or cell sorting

As reported previously^48^ with minor modification, cells were analyzed by FACSCantoII (BD Biosciences) and sorted using FACSAria Fusion (BD Biosciences) with a 70-μm nozzle. Data analysis was performed using FlowJo software version 10 (TreeStar). All cells were suspended in FACS flow buffer (BD Biosciences) and stained in the dark with appropriate antibodies and fixable Viability Dye eFluor506 (Invitrogen) for 15 min at 4 °C. Then, the cells were washed twice and used for subsequent experiments. The organ cells were gated on CD45.2^+^ for immune cells and CD3/CD19/NK1.1 negative (Lin^−^) for removing T, B and NK cells. FVD^−^Lin^−^CD45.2^+^CD14^−^BST2^−^MHCII^+^CD11c^high^ cells are a cDC population and were divided into XCR1^+^cDC1 and CD11b^+^cDC2. In vitro cDC1s derived from Flt3L-BMDC were gated as FVD^-^B220^–^MHCII^+^CD11c^+^ as total cDCs and then additionally gated as XCR1^+^. In the DC lineage, FVD^−^CD45.2^+^Lin^−^MHCII^−^CD11c^+^BST2^−^CD135^+^CD115^−^CD117^−/low^ is a line of pre-DCs. In pre-DCs, the subset of pre-cDC1 was SiglecH^−^Ly6C^−^, and the subset of pre-cDC2 was SiglecH^−^Ly6C^+^. pDCs were analyzed by gating on FVD^−^Lin^−^CD45.2^+^CD14^-^MHCII^+^B220^+^CD11c^int^ cells, NK cells were analyzed after pre-gating on FVD^−^CD45.2^+^CD3^−^. As reported previously^26^, EPs were gated as CD11c^+^CD117^+^, and then additionally gated as XCR1^−^CD24^−^, XCR1^−^CD24^+^ and XCR1^+^CD24^+^cDC1. For the surface staining of CD8^+^ T cell, single cells were stained with FVD^−^CD19^−^NK1.1^−^CD45.2^+^CD3^+^CD8α^+^. For the intracellular staining of CD8α^+^ T cells, single cells were already stimulated with cell activation cocktail (Biolegend) for 4 h, and then anti-IFN-γ, anti-perforin and anti-granzyme B were stained according to protocol of the Fixation/Permeabilization Kit (BD biosciences). The Foxp3/TF Staining Buffer Set (eBioscience) was used to stain Ki-67, a proliferation marker, in DC lineage cells. For TopBP1 intracellular staining, surface molecules were pre-stained and then fixed/permeabilized with Foxp3/TF Staining Buffer Set (eBioscience) for 20 min. The cells were washed and stained for 30 min at room temperature (RT) with control IgG or a rabbit polyclonal to TopBP1 (Abcam). The cells were washed, stained with FITC-conjugated anti-rabbit IgG (Biolegend) and analyzed by flow cytometry. Apoptosis analysis was conducted in pDC, pre-DCs and cDCs with the FITC Annexin V Apoptosis Detection Kit I (BD Biosciences), according to the manufacturer’s protocol.

### Tumor model and Flt3L immunotherapy

E.G7 and EL4 cells were cultured as reported previously^48^. Wild-type (WT), TopBP1^cKO^ and Batf3^KO^ mice were inoculated subcutaneously with 5 × 10^5^ of E.G7 cells in the right flank region. For tumor therapy, 3 μg of recombinant human Flt3L was intraperitoneally injected daily from day 8 of E.G7 inoculation. Tumor size was monitored every 3 days beginning 7 days after tumor injection. Tumor size was calculated as *V* = (short axis)^2^ × (long axis) × 1/2. BM cells, splenocytes and tumor-infiltrating lymphocytes (TILs) were analyzed by flow cytometry. For TIL isolation, tumors were homogenized by type IV collagenase and centrifuged with a discontinuous Percoll gradient (40 and 80%). Leukocytes were present in the interface of the gradient.

### CTL assay

As reported previously^48,49^ with minor modifications, CD8^+^ T cells isolated from spleen and TILs of TB mice were co-cultured with cell trace violet (Invitrogen)-labeled target cells (E.G7 cells) and carboxyfluorescein succinimidyl ester (Invitrogen)-labeled nonspecific target cells (EL4) for 24 h. After propidium iodide staining, cytotoxic T lymphocyte (CTL) activity was assessed by flow cytometry as the ratio of propidium iodide-cells in the cell trace violet- or carboxyfluorescein succinimidyl ester-labeled cells.

### Adoptive transfer

As reported previously^48^ with minor modifications, the BM cells (2 × 10^6^) obtained from WT (CD45.1^+^) and TopBP1^cKO^ (CD45.2^+^) mice were mixed at a 1:1 ratio and injected into mice through a tail vein of WT (CD45.1^+^CD45.2^+^) mice. After 7 days, pre-DCs in the BM and spleen differentiated from donor cells and cDCs in the spleen and lung were analyzed. After sorting pre-DCs (1 × 10^6^) in BM of WT (CD45.1^+^) and TopBP1^cKO^ (CD45.2^+^) mice, a 1:1 mixture was intravenously injected into recipient WT (CD45.1^+^CD45.2^+^) mice; 4 days later, the cDCs differentiated from donor cells were examined in the spleen. Pre-DCs were sorted from a minimum of ten mice for 1 AT experiment.

### In vitro generation of FL-BMDCs

As reported previously^48^, the tibias and femurs from killed mice were collected, and BM cells were isolated by flushing with PBS using a 1 ml syringe. Red blood cells were lysed with ACK lysis buffer for 5 min and then washed twice. BM cells isolated from mice were cultured as described previously^48,50,51^ with minor modifications. In brief, the BM cells were suspended at a density of 2 × 10^6^ per milliliter in cRPMI supplemented with 50 ng/ml recombinant human Flt3L, 10 mM HEPES buffer and 55 μM β-mercaptoethanol. The cells were plated at 1 ml per well on 24-well plates and were incubated at 37 °C with 5% CO_2_ for 9 days.

### Ex vivo culture of pre-DCs or EPs

Pre-DCs and EPs were isolated from the BM and spleens of WT (CD45.1^+^2^+^) and TopBP1^cKO^ (CD45.2^+^) mice, respectively, mixed at a 1:1 ratio and co-cultured with feeder cell Flt3L (50 ng/ml)-BMDCs (day 5) for 4–5 days. The differentiation into cDCs and subsets from donor-derived cells was analyzed. Pre-DCs were sorted from BM of both WT and TopBP1^cKO^ mice at a steady state. As reported previously^52,53^, recombinant Flt3L-Ig was intraperitoneally injected twice on days 0 and 3, and EPs were sorted from the spleen of injected mice on day 9.

### BrdU incorporation assay

Mice were intraperitoneally injected with BrdU (2 mg) and killed 2 h later. A BrdU incorporation assay was performed using a FITC BrdU kit according to the manufacturer’s protocol.

### Apoptosis analysis

Apoptosis analysis was conducted in DCs and precursors with the FITC Annexin V Apoptosis Detection Kit I according to the manufacturer’s protocol.

### Expression vectors and transfection

pcDNA3 vectors expressing PU.1, IRF8 and TopBP1 purchased from Addgene Inc. PcDNA3 vectors expressing PU.1, IRF8 and TopBP1 were transiently co-transfected into HEK293 cells using Lipofectamine 2000 (Invitrogen). After 18 h of transfection, the cells were collected and washed with PBS. The cells were then treated with trypsin, followed by PBS wash. Subsequently, the cells were lysed on ice for 30 min using lysis buffer containing 150 mM NaCl, 50 mM Tris–HCl (pH 7.4), 1 mM EDTA, 0.5% Triton X-100 and a protease inhibitor cocktail (Xpert). These cells were centrifuged at 16,000*g* for 10 min at 4 °C, and the lysate was collected.

### Co-IP and western blotting analysis

As described previously^54^ with minor modification. Splenocytes were washed with PBS and lysed in 150 mM NaCl, 50 mM Tris–HCl (pH 7.4), 1 mM EDTA, 1% Triton X-100 and protease inhibitor cocktail (Roche) on ice for 20 min and passed three times through a 28G syringe. The lysates were collected after centrifugation at 16,000*g* for 10 min at 4 °C. Then, anti-IgG, anti-PU.1 or anti-TopBP1 were added, respectively, and incubated at 4 °C overnight. After adding Pierce protein A/G magnetic beads the samples were incubated at RT for 1 h and then washed three times with wash buffer. The immunoprecipitated proteins were eluted by a sample buffer.

For western blotting analysis, proteins were separated on 8% SDS–polyacrylamide gels and then transferred by electrophoresis to PVDF membrane (Millipore). The membranes were blocked with 5% nonfat dry skim milk or BSA dissolved in TBST (20 mM Tris–HCl (pH 7.4), 150 mM NaCl, 0.05% Tween 20) for 1 h at RT and incubated overnight at 4 °C with optimal concentrations of primary antibodies diluted in 5% nonfat dry skim milk or BSA. Following four additional washes in TBST, the membranes were further incubated for 45 min with HRP-conjugated secondary antibodies. Bound antibodies were detected using chemiluminescent HRP substrate (Millipore).

### qRT–PCR

Total RNA was extracted from pre-DCs at a steady state from BM and spleens or EPs of Flt3L-injected mice using RNAiso Plus (TaKaRa). Reverse transcription was performed by Maxime RT PreMix. Real-time quantitative PCR was performed using iCycler real-time PCR machine and qGreen Q-PCR Master Mix (GenDEPOT) to measure the expression of genes under the following conditions: 40 cycles of 95 °C for 30 s, 60 °C for 30 s and 72 °C for 30 s. All reactions were independently repeated at least three times to verify reproducibility. Primer sequences for quantitative real-time polymerase chain reaction (qRT–PCR) amplification: *Zbtb46:* (F) AGAGAGCACATGAAGCGACA; (R) CTGGCTGCAGACATGAACAC *Spi1:* (F) CCCGGATGTGCTTCCCTTAT; (R) TCCAAGCCATCAGCTTCTCC *Id2:* (F) ATTCTGAACCGAGCCTGGTG; (R) TCACCGGACTGAAGGCTTTC *Irf8:* (F) CGTGGAAGACGAGGTTACGCTG; (R) GCTGAATGGTGTGTGTCATAGGC *Batf3:* (F) GTGTCCAGGGGTACATGTGG; (R) TCTGGATCCTCTGTCCTGGG *Irf4:* (F) GGATGACACACAGATGGCCA; (R) ATCCCTCCAGCTCCTGTCAT *Notch2:* (F) AACTGTCAGACCCTGGTGAAC; (R) CGACAAGTGTAGCCTCCAATC *Klf4:* (F) CGATGAACTGACCAGGCACTAC; (R) CCTCTTCATGTGTAAGGCAAGGTG *Relb:* (F) CCTCTCTTCCCTGTCACTAACGGTCTC; (R) ACGCTGCTTTGGCTGCTCTGTGATG *Nfil3:* (F) GAACTCTGCCTTAGCTGAGGT; (R) ATTCCCGTTTTCTTCTCCGACACG *Tdrd3:* (F) GCAGATGACGGATGGGCATA; (R) CACAGTGCCAGAGAGCTTCA *Thsd7b:* (F) CCTCTGACTGTCCAGCCTTG; (R) CATTCCTGGCCACCTCCAAT *Rchy1:* (F) GCAGTGAGAAGCCTGTCCTT; (R) ATCCTCAGGTGTTCACGCTG *Chrm3:* (F) ACTATGTGGCCAGCAATGCT; (R) CGGCTCGTTTTGTTGTTCGT *Ch25h:* (F) GCTCGTCCAGCTCCTAAGTC; (R) GGACGAGTTCTGGTGATGCA *Rab2a:* (F) AACTGTCAGACCCTGGTGAAC; (R) CTTCCTCCCATGCAACACCC *Fabp5:* (F) AGGATGGGAAGATGATCGTGTG; (R) GTTGCATTTGACCGCTCACT *Fam110b:* (F) AGCAGAGCAGGTGTTCTTTGT; (R) GCAGGAGGTGTCGTGAACTG *Ppm1h:* (F) CCAATTTCATGGGCGGCATC; (R) CTGCACTCCACCTCATCCTG *Ulk4*: (F) AGCAGCAGAAACGTCATGGA; (R) ATTCATTCAGCTGGCCTCCC *Mllt3:* (F) TGAATGTGACAAGATCGTGAACC; (R) GCGTCCAGCTGTTGTATCCT *Nsdhl:* (F) GTCCTCATGGCATTTTCGGC; (R) CAGCGGCTAAGATGTGTCCA *Nek1:* (F) TTCAGGCCTCAATGGAGCAG; (R) CTCTTGCTGTCTGCTCCACA *Bambi:* (F) TGAGAACAAGAGGCTGCAGG; (R) TTGCCGCATTTTGTCACAGG *Itih5:* (F) GAGCTCCTGAGGAATACGGC; (R) AACCAGCAGTCTACCTGTGC *Gapdh:* (F) TGTAGTTGAGGTCAATGAAGGG (R) ACATCGCTCAGACACCATG.

### ChIP-seq analysis

Pre-DCs from BM and CD24^+^ EPs from splenocytes of WT mice were sorted using the EZ-ChIP kit, following the manufacturer’s protocol. In brief, cells were suspended in RPMI, and 36% formaldehyde was added and incubated at RT for 6 min. After that, glycine was added, and the suspension was incubated for 5 min. After washing with cold PBS, cells were lysed in lysis buffer containing 1× protease inhibitor cocktail II, and chromatin was sheared by sonication to fragments of 200–500 bp. Cell lysates were cleared by centrifugation (14,000*g*, 4 °C, 10 min), and 1% of the extract was used as input, whereas the remaining lysate was used for immunoprecipitation with anti-TopBP1 or anti-H3K4me overnight at RT followed by a 1-h incubation with Pierce protein A/G magnetic beads. Beads were subsequently washed three times in lysis buffer, one time in lysis buffer with 500 mM NaCl, one time in wash buffer (10 mM Tris–Cl pH 8.0, 0.25 M LiCl, 1 mM EDTA, 0.5% NP-40 and 0.5% Na-deoxycholate) and one time in TE pH 8.0. Elution of immunoprecipitated complexes was achieved with 1% SDS, proteins were degraded with a final concentration of 1 mg/ml proteinase K (3 h, 42 °C), and crosslinks were reversed (8 h, 65 °C). DNA was purified by phenol-chloroform extraction by mixing 1:1 with a phenol/chloroform/isoamyl alcohol (25:24:1) solution, and phenol-chloroform residues were removed using phase lock gel tubes (Quantabio). DNA was then precipitated with pure ethanol at −20 °C, and chromatin immunoprecipitation sequencing (ChIP-seq) analysis was conducted by Macrogen. Raw data from the ChIP-seq analysis were deposited in NCBI Gene Expression Omnibus (GEO) under accession number GSE301438. ChIP-seq-based TF target genes were obtained from the Harmonizome database (https://maayanlab.cloud/Harmonizome), a resource developed by the Ma’ayan Laboratory at the Icahn School of Medicine at Mount Sinai.

### Bulk RNA-seq analysis

CD24^+^ EPs were isolated using an Aria sorter with an expanded DC lineage from the splenocytes of WT and TopBP1^cKO^ mice injected with recombinant Flt3L-Ig. RNA purification was performed using a RNeasy Mini Kit (QIAGEN) following the manufacturer’s protocols. The sequencing procedures from purified RNA were performed by ebiogen as reported previously^48^. The gene ontology analysis of genes downregulated in CD24^+^EP of the TopBP1^cKO^ was based on searches conducted in DAVID (http://david.abcc.ncifcrf.gov/). TFs’ target genes for further analyses were obtained from TF target gene resources provided by the Ma’ayan Laboratory (https://maayanlab.cloud) and from the TRRUST database (Transcriptional Regulatory Relationships Unraveled by Sentence-based Text mining; https://www.grnpedia.org/trrust/). Raw data from the bulk RNA sequencing (RNA-seq) analysis were deposited in NCBI GEO under accession number GSE300836.

### Confocal microscopy

As described previously^55^ with minor modifications, the proportion of CD11c^+^ cells among the splenocytes of WT or TopBP1^cKO^ mice treated with Flt3L-Ig was enriched by the Magnisort CD11c positive selection kit, and these cells were seeded on poly-L-lysine coverslips. The cells were fixed in 4% paraformaldehyde for 10 min at 37 °C and permeabilized in 0.1% Triton X-100 in PBS for 15 min at RT. After incubation with PBS containing 2% BSA for 1 h, samples were stained with the primary antibody overnight at 4 °C and then washed three times with PBS. Fluorescent dye-labeled secondary antibody incubation was performed for 45 min at RT in the dark, and nuclei were stained with Hoechst for 10 min. The cover glasses were mounted onto glass slides and visualized using a Zeiss LSM700 microscope (Zeiss). The acquired images were subsequently processed with Zen software (Zeiss). The following antibodies were used: TopBP1, PU.1, ICSBP Alexa Fluor 488 and IRF8. The secondary antibodies were Alexa Fluor 488 anti-mouse IgG and Alexa Fluor 647 anti-rabbit.

### Quantification and statistical analysis

**Fig. 1:**
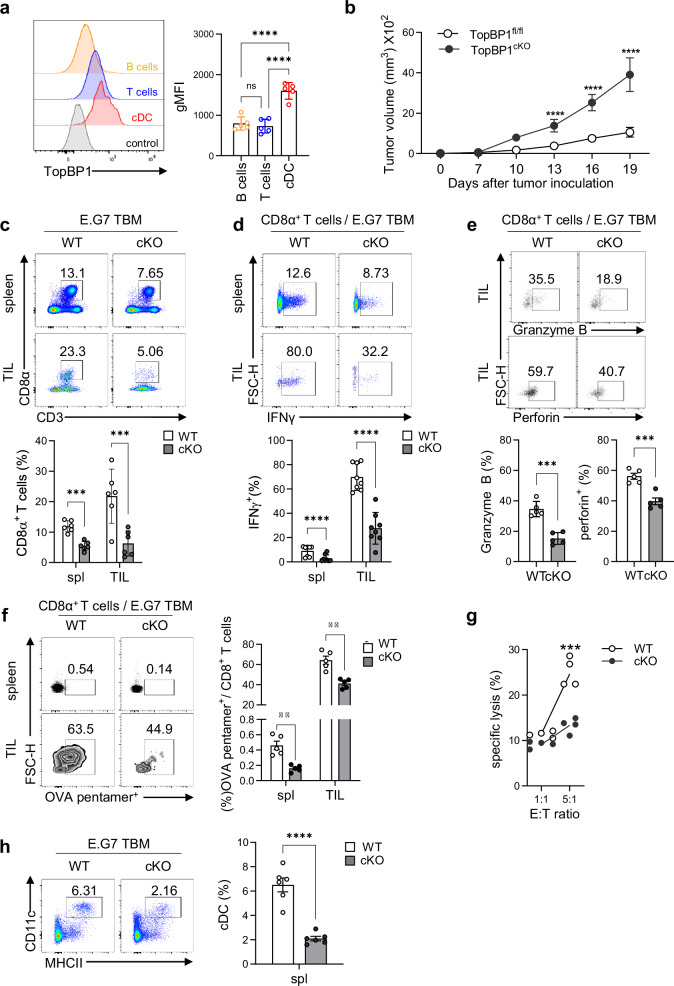
Rapid tumor growth in TopBP1^cKO^ mice due to impaired anti-tumor immunity. **a** Expression levels of TopBP1 in B cells, T cells and cDCs. **b** E.G7 tumor growth in WT and TopBP1^cKO^ mice. *n* = 8 per group. **c** CD8^+^T cells in the spleens and TILs of E.G7-TBM. *n* = 6 per group. **d** IFNγ^+^ effector CD8^+^ T cells in TILs of E.G7-TBM. *n* = 8 per group. **e** Granzyme B- and perforin-expressing CD8^+^ T cells in TILs of WT and TopBP1^cKO^ E.G7-TBM. *n* = 5 per group. **f** OVA-specific CD8α⁺ T cells in the spleens and TILs of WT and TopBP1^cKO^ E.G7-TBM. *n* = 5 per group. **g** CTL activity in the TILs of WT and TopBP1^cKO^ E.G7-TBM. *n* = 4 per group. **h** The frequency of total cDCs in the spleens of WT and TopBP1^cKO^ E.G7-TBM. *n* = 6 per group. An unpaired one-way ANOVA with Tukey’s multiple comparisons test was used for **a** two-way ANOVA with Turkey’s multiple comparisons test was used for **b**–**d** and **f**–**g**), and an unpaired *t*-test with Welch’s correction was used were used for statistical analysis in **e** and **h**. **P < 0.01, ***P < 0.001, ****P < 0.0001; the error bars indicate mean ± s.e.m.

**Fig. 2:**
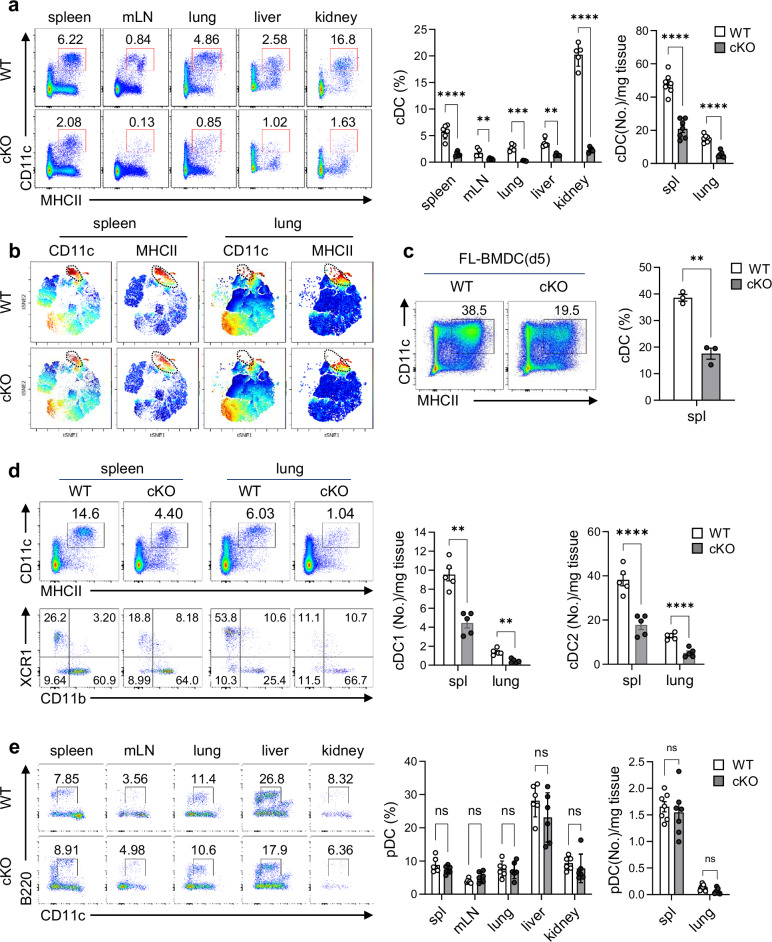
Conditional depletion of TopBP1 leads to cDC deficiency in mice. **a** Total cDCs in lymphoid organs (spleen and LN) and nonlymphoid organs (lung, liver and kidney) of WT and TopBP1^cKO^ mice at a steady state. *n* = 6 per group. **b** t-SNE plot of cytometry analysis of live immune cells in the spleens and lungs of WT and TopBP1^cKO^ mice at a steady state. (gated on FVD^−^Lin^−^BST2^−^CD45^+^) **c** cDC populations on day 5 of Flt3L (50 ng/ml)-BMDC generation in WT and TopBP1^cKO^ mice. *n* = 3 per group. **d** cDC subsets in the spleens and lungs of WT and TopBP1^cKO^ mice. *n* = 5 per group. **e** pDCs in lymphoid organs and nonlymphoid organs of WT and TopBP1^cKO^ mice at a steady state. *n* = 6 per group. Unpaired multiple *t*-tests for **a**, **d** and **e** and unpaired *t*-test with Welch’s correction for **c** were used for statistics. **P < 0.01, ***P < 0.001, ****P < 0.0001; ns not significant, the error bars indicate mean ± s.e.m.

**Fig. 3:**
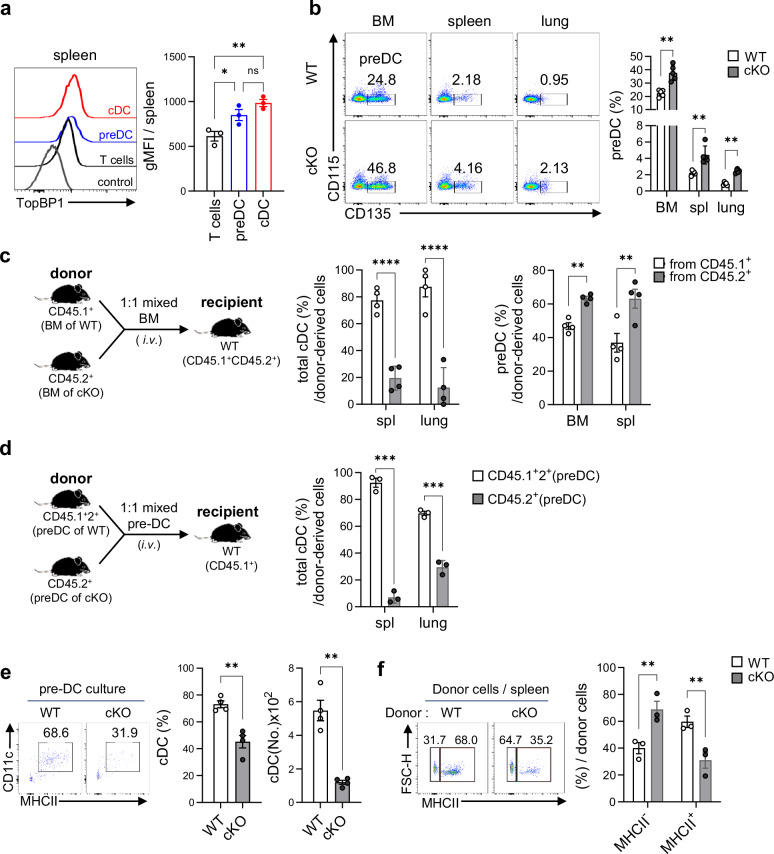
TopBP1 is an intrinsic factor required for pre-DC differentiation into cDCs. **a** Expression levels of TopBP1 in pre-DCs and cDCs were compared with that of T cells as a positive control in the spleens of WT mice. *n* = 3 per group. **b** Populations of pre-DCs in the spleens, BM and lungs of WT and TopBP1^cKO^ mice at a steady state. *n* = 5 per group. **c**, Total BM cells of WT (CD45.1^+^) and TopBP1^cKO^ (CD45.2^+^) mice were mixed at a 1:1 ratio and injected intravenous (i.v.) into recipient WT (CD45.1^+^2^+^) mice. After 7 days, cDCs and pre-DCs differentiated from donor BM cells were assessed. *n* = 4 per group. **d** Pre-DCs sorted from the BM of WT (CD45.1^+^2^+^) and TopBP1^cKO^ (CD45.2^+^) mice were mixed at a 1:1 ratio and injected i.v. into recipient WT (CD45.1^+^) mice. After 5 days, cDCs differentiated from donor pre-DCs were assessed in the spleen and lung. *n* = 3 per group, with ten mice used per replicate. **e** Pre-DCs sorted from the BM of WT (CD45.1^+^2^+^) and TopBP1^cKO^ (CD45.2^+^) mice were cultured with CD45.1^+^ feeder cells (50 ng/ml Flt3L-BMDC, d5). After 4 days, cDCs differentiated from donor pre-DCs were assessed. *n* = 4 per group, with five mice used per replicate. **f** Pre-DCs sorted from the BM of WT (CD45.1^+^2^+^) and TopBP1^cKO^ (CD45.2^+^) mice were mixed at a 1:1 ratio and injected i.v. into recipient WT (CD45.1^+^) mice. After 2 days, MHCII expression levels in donor cells were assessed in the spleen of recipient mice. *n* = 3 per group, with six mice used per replicate. Unpaired one-way ANOVA with Tukey’s multiple comparisons test for **a** two-way ANOVA with Tukey’s multiple comparisons test for **b**–**d** unpaired *t*-test with Welch’s correction for **e** and two-way ANOVA with Bonferroni for multiple comparisons for **f** were used to measure significance. *P < 0.05, **P < 0.01, ***P < 0.001, ****P < 0.0001, ns not significant, the error bars indicate mean ± s.e.m.

**Fig. 4:**
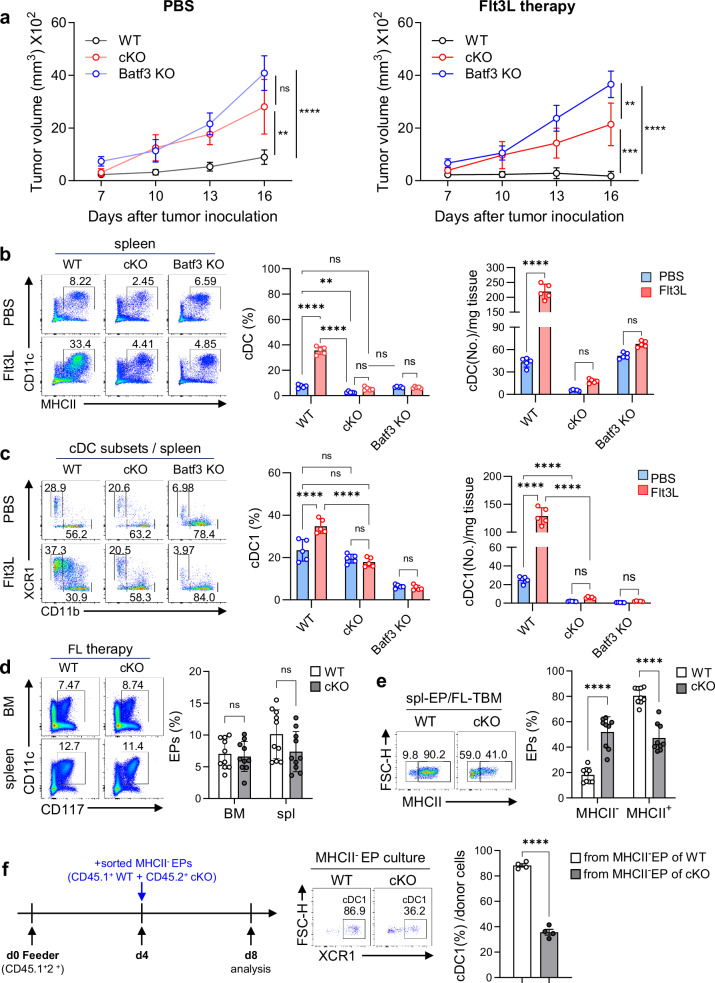
Flt3L-based tumor immunotherapy was not effective in TopBP1^cKO^ TBM due to impairment of cDC1 development. **a** Tumor growth was monitored in WT, TopBP1^cKO^ and Batf3^KO^ E.G7-TBM treated with Flt3L as a tumor immunotherapy. Recombinant Flt3L (3 µg) was administered i.p. daily from day 9 to day 18 after tumor injection. *n* = 8 per group. **b** Analysis of cDCs on day 16 in the spleens of WT, TopBP1^cKO^ and Batf3^KO^ E.G7-TBM treated with Flt3L. *n* = 5 per group. **c**, Analysis of cDC1 subset on day 16 in the spleens of WT and TopBP1^cKO^ E.G7-TBM treated with Flt3L. *n* = 5 per group. **d** Total EPs (CD117^+^CD11c^+^) were assessed in the BM and spleens of WT, TopBP1^cKO^ and Batf3^KO^ E.G7-TBM treated with Flt3L. *n* = 10 per group. **e** MHCII^+^ population in the splenic EPs of Flt3L-treated WT and TopBP1^cKO^ E.G7-TBM. *n* = 9 per group. **f** MHCII^-^ EPs were sorted from WT (CD45.1^+^) and TopBP1^cKO^ (CD45.2^+^) BM cells cultured for 2 days with Flt3L (50 ng/ml) and then further cultured with FL-BMDCs (day 4) as feeder cells (CD45.1^+^2^+^). Four days later, the population of cDC1s differentiated from MHCII^−^ EPs were assessed. *n* = 4 per group. Unpaired two-way ANOVA with Tukey for multiple comparisons for **a**–**e** and an unpaired *t*-test with Welch’s correction for **f** were used to measure significance. **P < 0.01, ***P < 0.001, ****P < 0.0001, ns not significant, the error bars indicate mean ± s.e.m.

**Fig. 5:**
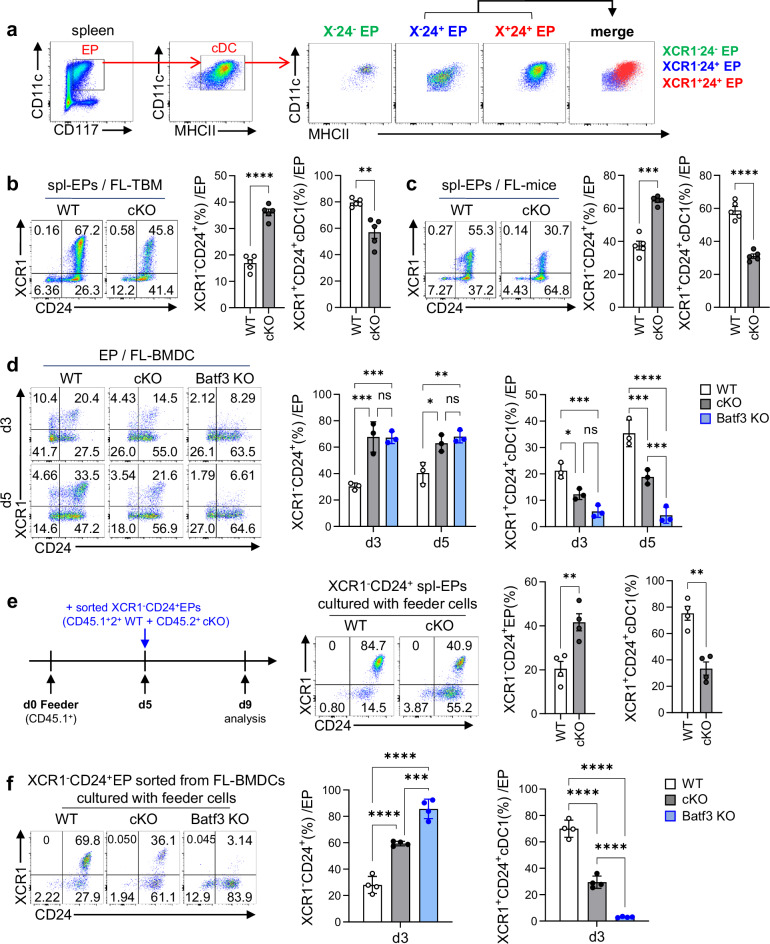
TopBP1 is essential for differentiation of XCR1^-^CD24^+^ EPs to XCR1^+^CD24^+^ cDC1s. **a** The cDC gate in the splenic EPs of Flt3L-treated WT TBM was further examined for the expression of cDC1 markers, CD24 and XCR1, and the three distinct populations were re-plotted based on MHCII and CD11c expression levels. **b** Splenic EPs from the Flt3L-treated WT and TopBP1^cKO^ TBM were analyzed based on the expression of XCR1 and CD24. *n* = 5 per group. **c** Splenic EPs from WT and TopBP1^cKO^ mice treated with Flt3L-Ig on day 0 and day 3 were analyzed on day 9 based on the expression of XCR1 and CD24. *n* = 5 per group. **d** Total EPs of WT, TopBP1^cKO^ and Batf3^KO^ mice were further examined based on the expression levels of XCR1 and CD24, which are cDC1 markers, during the FL-BMDC cultures on day 3 and 5. *n* = 3 per group. **e** XCR1^−^CD24^+^ EPs were sorted on day 9 from total splenic EPs of WT (CD45.1^+^2^+^) and TopBP1^cKO^ (CD45.2^+^) mice treated with Flt3L-Ig on day 0 and day 3, and equal numbers of these EPs were cultured with CD45.1^+^ feeder cells (FL-BMDCs on day 5). Four days later, the population of cDC1s differentiated from XCR1^−^CD24^+^ EPs was assessed. *n* = 4 per group. **f** XCR1^−^CD24^+^ EPs were sorted from FL-BMDC cultures on day 5 of WT (CD45.1^+^2^+^), TopBP1^cKO^ (CD45.2^+^) and Batf3^KO^ (CD45.2^+^) mice, respectively. Equal numbers of sorted cells were cultured with CD45.1^+^ feeder cells (FL-BMDCs on day 5). After 5 days, the population of XCR1^−^CD24^+^EPs and XCR1^+^CD24^+^ cDC1s differentiated from the cultures were assessed. *n* = 4 per group. Unpaired *t*-test with Welch’s correction for **b**, **c** and **e** unpaired two-way ANOVA with Tukey for post-test for **d** and unpaired one-way ANOVA with Tukey for post-test for **f** were used to measure significance. *P < 0.05, **P < 0.01, ***P < 0.001, ****P < 0.0001; ns not significant; the error bars indicate mean ± s.e.m.

**Fig. 6:**
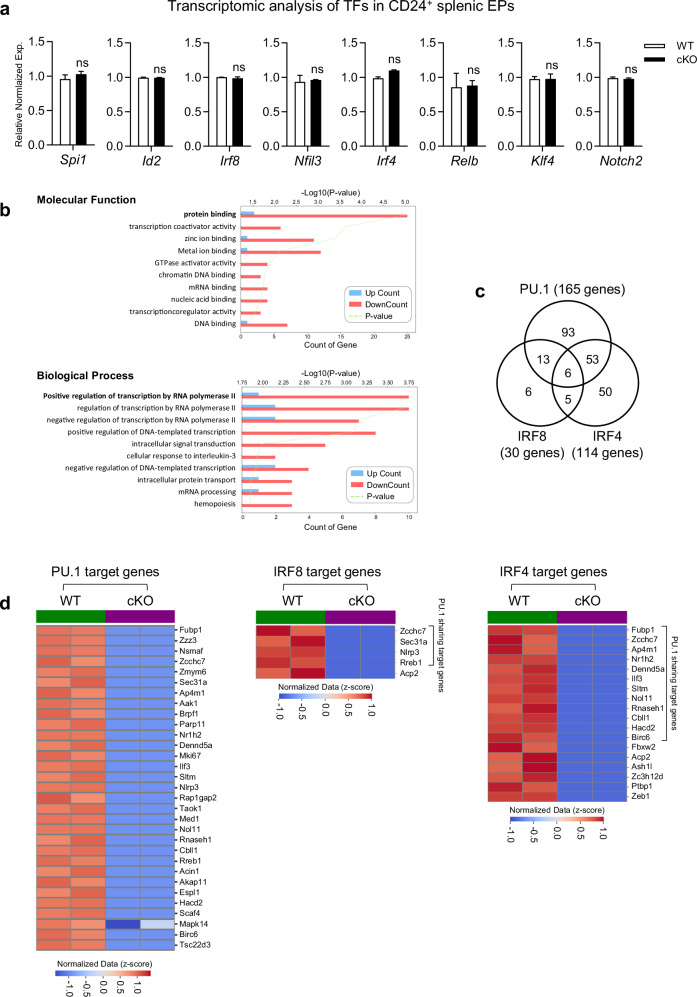
Depletion of TopBP1 in cDCs leads to reduced expression of target genes regulated by PU.1, IRF8 and IRF4. **a** The relative expression levels of key TFs involved in cDC differentiation were assessed basis on bulk RNA-seq transcriptomics of CD24^+^ splenic EPs collected on day 9 from WT and TopBP1^cKO^ mice injected i.p. with Flt3L-Ig on day 0 and day 3. An unpaired *t*-test was used to measure significance. ns, not significant; the error bars indicate mean ± s.e.m. **b** The −log_10_ *P* values of pathways enriched in the DAVID-based gene ontology analysis of downregulated genes (fold change < 0.5, *P* value <0.05) from the transcriptomics of CD24^+^ splenic EPs in TopBP1^cKO^ mice compared with those of WT mice. **c** The Venn diagram shows the numbers of PU.1, IRF8 and IRF4 target genes downregulated (fold change <0.5, FDR <0.05) in CD24^+^ splenic EPs of TopBP1^cKO^ mice compared with those of WT mice with the bulk RNA-seq transcriptomic data described in **a**. The number of genes in each category is indicated. **d** A heat map showing the relative expression of genes identified as targets of PU.1, IRF8 and IRF4, which are reduced to 0.1-fold or less (FDR <0.05) in TopBP1^cKO^ EPs compared with WT EPs based on transcriptomics.

**Fig. 7:**
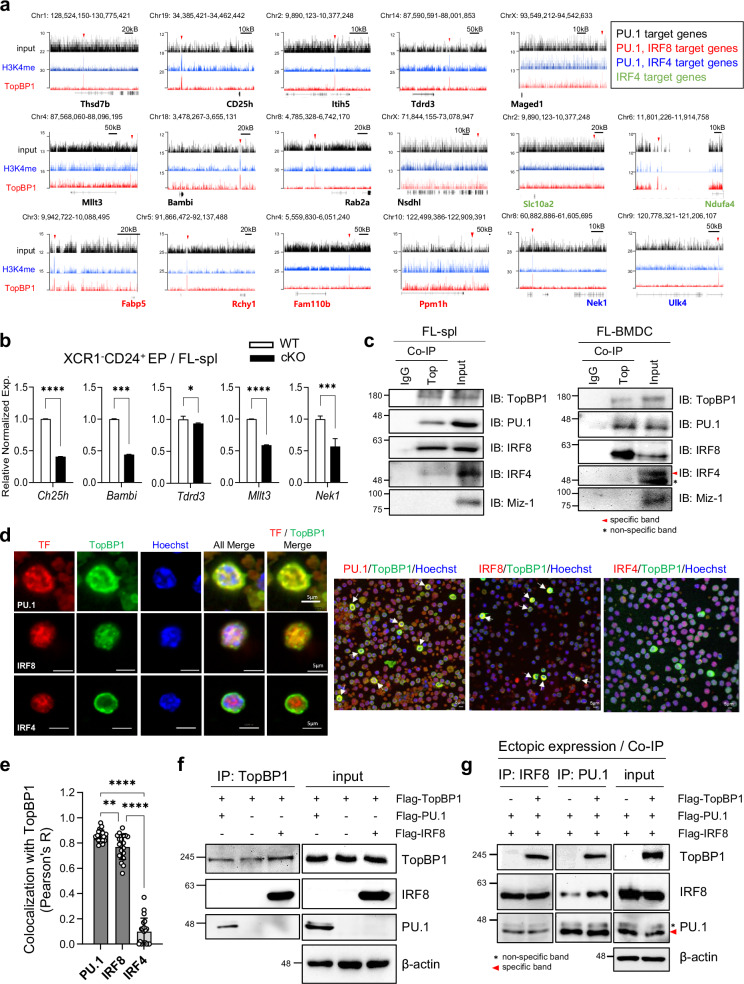
TopBP1 drives cDC development via interaction with both PU.1 and IRF8. **a** ChIP-seq analysis on CD24^+^ splenic EPs, collected on day 9 from WT mice injected with Flt3L-Ig on days 0 and 3, using anti-TopBP1 and H3K4me antibodies. Normalized ChIP-seq binding tracks are shown for the genes identified, with differential TopBP1 binding regions marked by red arrows. **b** mRNA expression levels of the target genes, identified by ChIP-seq analysis in **a** were examined by qRT–PCR in XCR1^−^CD24^+^ splenic EPs of WT and TopBP1^cKO^ mice. **c** Co-IP analysis of PU.1 and IRF8 using an anti-TopBP1 antibody on CD24^+^ splenic EPs from WT mice injected with Flt3L-Ig (left) and on FL-BMDCs (right), followed by immunoblot (IB) analysis with appropriate antibodies. **d** Confocal imaging of total CD11c^+^ cells enriched from the splenocytes of WT mice injected with Flt3L-Ig, following staining with fluorescence-labeled antibodies against TopBP1, PU.1, IRF8 and IRF4. Hoechst serves as a DNA stain. **e** Colocalization of TopBP1 with PU.1, IRF8 or IRF4 was quantified using the Coloc 2 plugin in Fiji program. Pearson’s correlation coefficients (below threshold) were calculated from 20 individual cells (regions of interest, ROIs) per group. Statistical analysis was performed using one-way ANOVA followed by Tukey’s multiple comparison test (**P < 0.01, ****P < 0.0001). The error bars represent mean ± s.d. **f** Co-IP analysis of PU.1 and IRF8 with TopBP1 using an anti-TopBP1 antibody in HEK293 cells co-transfected with plasmids expressing human PU.1, IRF8 and TopBP1. **g** Co-IP analysis of PU.1 and IRF8 in HEK293 cells co-transfected with plasmids expressing mouse PU.1, IRF8 and either with or without TopBP1, using anti-PU.1 antibodies.

Statistical analysis was performed using Prism 10 software (GraphPad). For group statistics comparison value, one-way analysis of variance (ANOVA) with a Tukey’s or Dunnett’s multiple comparisons test or two-way ANOVA with a Bonferroni’s multiple comparisons test was employed, with significance indicated as *P < 0.05, **P < 0.01, ***P < 0.001, ****P < 0.0001, and ‘ns’ denotes nonsignificant differences, in Figs. 1–7.

## Results

### TopBP1^cKO^ mice exhibited rapid tumor growth due to impaired anti-tumor immunity

Although TopBP1 is essential for V(D)J recombination and development of T and B cells^19^, its expression level was significantly higher in cDCs than in T and B cells (Fig. 1a). TopBP1 expression was further confirmed at the mRNA level during the in vitro generation of Flt3L-mediated BM-derived DCs (FL-BMDCs) (Supplementary Fig. 1a). We generated DC-specific TopBP1-depleted (TopBP1^cKO^; CD11c-cre × TopBP1^fl/fl^) mice (Supplementary Fig. 1b) and examined tumor growth. TopBP1^cKO^ tumor-bearing mice (TBM) exhibited notably faster tumor progression (Fig. 1b) and splenomegaly (Supplementary Fig. 1c) compared with littermate control (TopBP1^fl/fl^ WT) TBM. The population (Fig. 1c) and number (Supplementary Fig. 1d) of CD8α^+^ T cells were significantly reduced in the spleen and TILs of TopBP1^cKO^ TBM compared with WT TBM. Effector CD8^+^ T cells, secreting IFNγ (Fig. 1d), perforin and granzyme B (Fig. 1e), were also markedly decreased in the TILs of TopBP1^cKO^ TBM. In addition, the population of OVA-specific CD8^+^ T cells, critical for anti-tumor immunity against OVA-expressing E.G7 tumors, was significantly lower in both the spleen and TILs of TopBP1^cKO^ mice (Fig. 1f). CTL responses in TILs were also impaired in TopBP1^cKO^ TBM (Fig. 1g). Furthermore, the frequency and number of CD4^+^ T cells (Supplementary Fig. 1e) and IFNγ^+^CD4^+^ T cells in the spleen, inguinal LN and TILs (Supplementary Fig. 1f) were significantly reduced in TopBP1^cKO^ TBM. By contrast, NK cell populations remained unaffected in the spleen and inguinal LN but were instead increased in the TILs of TopBP1^cKO^ TBM (Supplementary Fig. 1g). In further analysis of the reduction of effector T cells in TopBP1^cKO^ mice, we found no differences in T cell development in the thymus between WT and TopBP1^cKO^ mice (Supplementary Fig. 2a), and the total number of thymic CD3⁺ T cells was also comparable between the two groups (Supplementary Fig. 2b). In addition, the frequency of apoptotic CD8α⁺ and CD4⁺ T cells in the spleen showed no differences between the two groups (Supplementary Fig. 2c). These results suggest that the reduced numbers of effector T cells observed in TopBP1^cKO^ TBMs are not due to defects in T cell development or increased apoptosis but are probably a secondary effect of altered cDC population. Supporting this, the cDC population in the spleen was significantly reduced in TopBP1^cKO^ TBM compared with WT TBM (Fig. 1h). Overall, these findings suggest that the rapid tumor growth in TopBP1^cKO^ mice results from impaired anti-tumor immunity due to defects in TopBP1-mediated cDC development.

### Reduced cDC levels in lymphoid and nonlymphoid organs of TopBP1^cKO^ mice

We examined the DC population at a steady state based on the gating strategy illustrated in Supplementary Fig. 3a. Intriguingly, the population of cDCs was significantly reduced in TopBP1^cKO^ mice compared with WT littermates in lymphoid organs (spleen and mesenteric LN) and nonlymphoid (lung, liver and kidney) organs (Fig. 2a). Compared with WT controls, the t-distributed stochastic neighbor embedding (t-SNE) plots of TopBP1^cKO^ splenocytes showed a clear decrease in cells expressing CD11c and MHCII (Fig. 2b). Consistent with the results in TopBP1^cKO^ mice, cDC (CD11c^+^MHCII^+^) populations were significantly reduced in in vitro-generated TopBP1^cKO^ FL-BMDCs compared with that in WT FL-BMDCs (Fig. 2c). In further subset analysis, the numbers of both XCR1^+^CD11b^−^ cDC1s and XCR1^−^CD11b^+^ cDC2s were also significantly reduced in TopBP1^cKO^ mice (Fig. 2d). However, the population and number of pDCs remained unchanged in both lymphoid and nonlymphoid organs in both TopBP1^cKO^ and WT mice (Fig. 2e). These results suggest that TopBP1 plays a crucial role in cDC development but is little involved in pDC development.

### TopBP1 is intrinsically required for the differentiation of pre-DCs into cDCs

We considered three possible reasons for the reduction of cDCs in TopBP1^cKO^ mice: (1) impairment of cDC differentiation, (2) impairment of pre-DC/cDC proliferation and/or (3) cDC apoptosis increased in TopBP1^cKO^ mice due to accumulation of DNA damages. Normally, pre-DCs (Lin^−^BST2^−^CD117^−/int^MHCII^−^CD11c^+^CD115^−^CD135^+^) express TopBP1 at levels comparable to those observed in cDCs (Fig. 3a). In TopBP1^cKO^ mice, pre-DCs accumulated significantly in the BM and other organs compared with WT mice (Fig. 3b). It is well known that pre-DCs comprise pre-cDC1s (SiglecH^−^Ly6C^−^) and pre-cDC2s (SiglecH^−^Ly6C^+^) and then differentiate into cDC1 and cDC2 subsets, respectively^56^. Analysis of pre-DC subsets revealed no significant differences in the population of pre-cDC1 and pre-cDC2 between WT and TopBP1^cKO^ mice (Supplementary Fig. 3b). These results imply that TopBP1 is probably involved in the differentiation of pre-DCs into cDCs, regardless of final fate. Proliferation capacity was assessed based on the expression of the proliferation marker Ki-67 and BrdU incorporation. Although the proportion of Ki-67⁺ cells in BM pre-DCs was comparable between WT and TopBP1^cKO^ mice (Supplementary Fig. 4a), the proportions of Ki-67⁺ cells in splenic cDC1s, cDC2s and pDCs were increased in TopBP1^cKO^ mice compared with WT (Supplementary Fig. 4b). To validate this result more precisely, we analyzed BrdU incorporation, which revealed a significant increase in BrdU⁺ cells in both BM and splenic pre-DCs and cDCs of TopBP1^cKO^ mice compared with WT controls (Supplementary Fig. 4c). In addition, there was no significant difference in pre-DC apoptosis rates in the BM and spleen (Supplementary Fig. 5a) or in cDC subsets in the spleen and lungs (Supplementary Fig. 5b) between WT and TopBP1^cKO^ mice. DNA damage analysis by staining with γH2AX, a DNA damage marker, showed no significant difference in the extent of DNA damage accumulation between WT and TopBP1^cKO^ mice in BM-derived pre-DCs (Supplementary Fig. 6a), whereas in pre-DC subsets, there was a trend toward even further reduction in DNA damage accumulation in the TopBP1^cKO^ mice (Supplementary Fig. 6b). In addition, no clear difference in DNA damage accumulation was observed between WT and TopBP1^cKO^ mice in the cDC subsets (Supplementary Fig. 6c). Overall, our findings suggest that the significant reduction in the cDC population observed in TopBP1^cKO^ mice is primarily due to an impaired differentiation process from pre-DCs to cDCs rather than reduced cDC proliferation capacity, increased DNA damage accumulation or increased cDC apoptosis during cDC development in TopBP1^cKO^ mice.

Next, to determine whether TopBP1 is an intrinsic or extrinsic factor in cDC development, WT (CD45.1^+^) and TopBP1^cKO^ (CD45.2^+^) BM cells were mixed at a 1:1 ratio and adoptively transferred into WT(CD45.1^+^CD45.2^+^) recipient mice. The cDC population derived from TopBP1^cKO^ BM cells was significantly reduced compared with that derived from WT BM cells, whereas the pre-DC population derived from TopBP1^cKO^ BM cells was increased (Fig. 3c). Furthermore, in the adoptive transfer of pre-DC mixtures obtained from the BM of WT (CD45.1^+^2^+^) and TopBP1^cKO^ (CD45.2^+^) mice into WT (CD45.1^+^) recipient mice, the population of cDCs derived from TopBP1^cKO^ pre-DCs was substantially lower than that derived from WT pre-DCs (Fig. 3d). When the pre-DCs sorted from the BM of WT and TopBP1^cKO^ mice were cultured in the presence of feeder cells (FL-BMDCs on day 5), the cDC population derived from TopBP1^cKO^ pre-DCs was also markedly reduced compared with cDCs derived from WT pre-DCs (Fig. 3e). These data suggest that TopBP1 is an intrinsic factor in the differentiation of pre-DCs into cDCs. Another interesting observation is that 2 days after adoptive transfer of pre-DCs, the MHCII^+^ population among cDCs was markedly reduced in TopBP1^cKO^ pre-DC-derived cells compared with WT pre-DC-derived cells (Fig. 3f). This implies that TopBP1 is involved in the transition from MHCII^−^ to MHCII^+^ during cDC differentiation. This observation is further delineated in the Discussion.

### Flt3L-based tumor immunotherapy was not effective in TopBP1^cKO^ TBM due to impairment of cDC1 development

Flt3L is a growth factor widely used in tumor immunotherapy to increase immune cells^25^. It was recently reported that Flt3L amplifies small amounts of EPs (CD11c^+^CD117^+^) in the BM and then promotes further differentiation of amplified EPs into cDC1s^26^, inducing anti-tumor immunity. Consistent with a previous report, the EP population dramatically increased in the BM of Flt3L-expressing B16F10 melanoma TBM compared with B16F10 TBM or GM-CSF-expressing B16F10 TBM (Supplementary Fig. 7a). When pre-DCs and EPs obtained from the BM of control and Flt3L-injected mice, respectively, were mix-cultured with feeder cells, cDC1s were more efficiently differentiated from Flt3L-derived EPs than from pre-DCs (Supplementary Fig. 7b).

To determine whether the reduced anti-tumor immunity in TopBP1^cKO^ TBM could be restored by Flt3L immunotherapy, Flt3L was administered intraperitoneally (i.p.) daily to E.G7-TBM. In WT TBM, Flt3L immunotherapy effectively inhibited tumor growth, but this suppression was absent in TopBP1^cKO^ TBM, mirroring the observations in cDC1-deficient Batf3^KO^ TBM (Fig. 4a). Flt3L administration resulted in a significant increase in the population and number of cDCs in WT TBM; however, this marked enhancement was not observed in TopBP1^cKO^ and Batf3^KO^ TBM (Fig. 4b). As expected, the majority population among the expanded cDCs in the spleen of Flt3L-injected WT TBM was cDC1s, whereas in TopBP1^cKO^ TBM, the cDC1 population remained unchanged, with only a marginal increase in cell numbers even after Flt3L administration (Fig. 4c). Moreover, antigen-specific CTL responses were not effectively induced in TopBP1^cKO^ TBM compared with WT TBM (Supplementary Fig. 7c). However, there was no significant difference in the populations of total EPs in the BM and spleen between WT and TopBP1^cKO^ TBM receiving Flt3L immunotherapy (Fig. 4d). Further analysis of total EPs based on MHCII expression revealed a significant reduction in the MHCII^+^ population and a notable accumulation of the MHCII^−^ population in TopBP1^cKO^ TBM compared with WT TBM (Fig. 4e). This observation is also delineated in the Discussion. MHCII^−^ EPs were sorted from the spleens of Flt3L-injected WT (CD45.1^+^) or TopBP1^cKO^ (CD45.2^+^) mice and cultured in the presence of feeder cells (CD45.1^+^2^+^) to induce differentiation, as illustrated in Fig. 4f. MHCII^−^ EPs from TopBP1^cKO^ failed to properly differentiate into cDC1s compared with those from WT MHCII^−^ EPs (Fig. 4f). These results indicate that the inefficient tumor suppression by Flt3L immunotherapy in TopBP1^cKO^ TBM is probably due to an impairment in the differentiation process from EPs into cDC1s. By contrast, ratio of the splenic cDC2 populations between WT and TopBP1^cKO^ mice showed no clear difference at a steady state. However, in Flt3L-treated E.G7-TBMs, WT mice exhibited a marked increase in the cDC1 population, resulting in a relatively reduced proportion of cDC2s. In TopBP1^cKO^ mice, cDC1 differentiation was severely impaired, leading to a relatively increased proportion of cDC2s (Supplementary Fig. 7d, left). These results suggest that the cDC2 population in TBMs is not substantially affected by Flt3L-treatment, regardless of TopBP1 status. However, the absolute cell number of splenic cDC2 was substantially reduced in TopBP1^cKO^ mice compared with WT mice in both PBS- and FL-treated groups (Supplementary Fig. 7d, right), suggesting that TopBP1 is also essential for cDC2 development.

### TopBP1 plays a crucial role in the differentiation process from XCR1^−^CD24^+^ EPs to XCR1^+^CD24^+^cDC1s in Flt3L-mediated cDC1 development

The population of cDCs within EPs was significantly reduced in TopBP1^cKO^ Flt3L-TBM compared with WT Flt3L-TBM (Supplementary Fig. 7e). Since EPs are defined based solely on the expression of CD11c and CD117 (c-kit), they constitute a heterogeneous population encompassing both DC progenitors and fully differentiated cDCs^26^. To determine at which stage the differentiation from EPs to cDCs is impaired in TopBP1^cKO^ mice, we analyzed splenic EPs from Flt3L-injected mice using XCR1 and CD24, which are established surface markers for cDC1s^57^. Within the splenic EPs of WT Flt3L-TBM, the cDC gate was further examined for cDC1 markers, revealing three distinct populations: XCR1^−^CD24^−^, XCR1^−^CD24^+^ and XCR1^+^CD24^+^ (cDC1). Given the lower expression levels of MHCII and CD11c in the XCR1⁻CD24⁺ population, we hypothesized that this subset serves as a precursor to XCR1⁺CD24⁺ cDC1s (Fig. 5a). Although the total EP populations were comparable between WT and TopBP1^cKO^ TBM injected with Flt3L (Fig. 4d), a notable finding was the significant increase in the XCR1⁻CD24⁺ population, accompanied by a marked decrease in the XCR1⁺CD24⁺cDC1 population in the splenic EPs of TopBP1^cKO^ TBM compared with those of WT TBM (Fig. 5b). This accumulation of XCR1^−^CD24^+^ cDC1 precursors was similarly observed in tumor-free TopBP1^cKO^ mice injected with Flt3L (Fig. 5c). Similarly, in FL-BMDCs generated in vitro, the XCR1^-^CD24^+^ population accumulated, whereas the fully differentiated XCR1^+^CD24^+^ cDC1 population was significantly reduced in TopBP1^cKO^ FL-BMDCs compared with WT FL-BMDCs (Fig. 5d). Batf3^KO^ mice, in which differentiation into cDC1s is blocked, were used as a control. Differentiation into XCR1⁻CD24⁺EP cells in TopBP1^cKO^ mice was comparable to that observed in Batf3^KO^ mice (Fig. 5d). To determine whether XCR1⁺CD24⁺ cDC1s differentiate from XCR1⁻CD24⁺ EPs, XCR1⁻CD24⁺ EPs were sorted from the spleens of Flt3L-injected mice and cultured with feeder cells. The generation of XCR1^+^CD24^+^ cDC1s was markedly reduced in cultures derived from TopBP1^cKO^ EPs compared with those from WT EPs (Fig. 5e). Consistent with these results, when XCR1^−^CD24^+^ EPs sorted from the FL-BMDCs were cultured with feeder cells (CD45.1^+^), the development of XCR1^+^CD24^+^ cDC1s was also dramatically reduced in TopBP1^cKO^ EPs—similar to what was observed in Batf3^KO^ mice—compared with WT EPs (Fig. 5f). Overall, these results indicate that TopBP1 plays a crucial role in the differentiation of XCR1^−^CD24^+^EPs into XCR1^+^CD24^+^ cDC1s. Meanwhile, XCR1^−^CD24^−^ cells, probably corresponding to cDC2s, represented a minor population among the EPs (Fig. 5a), and MHCII^+^ population within these cells was also significantly reduced in TopBP1^cKO^ EPs compared with WT EPs (Supplementary Fig. 8a). Even when further cultured with feeder cells, these XCR1^−^CD24^−^ WT EPs did not express XCR1 within the DC gate (Supplementary Fig. 8b).

### Reduced expression of DC-related TF target genes in the pre-DCs and EPs of TopBP1^cKO^ mice

As TopBP1^cKO^ mice showed only cDC deficiency without affecting the differentiation of pDCs, we investigated the expression of zbtb46, a key TF for the development of cDCs^58^, in the pre-DC and cDCs of TopBP1^cKO^ mice. However, the zbtb46 expression was not affected by TopBP1 depletion in pre-DCs/cDCs or in EPs in the BM and spleens of WT and TopBP1^cKO^ mice (Supplementary Fig. 9a). We further examined the expression of well-established TFs involved in cDC differentiation using qRT–PCR in pre-DCs isolated from the BM of WT and TopBP1^cKO^ mice. Most TFs did not show any significant reduction in expression in the pre-DCs of TopBP1^cKO^ mice; however, some TFs, including *Batf3*, *IRF4* and *Notch2*, exhibited increased expression in TopBP1^cKO^ pre-DCs compared with WT pre-DCs (Supplementary Fig. 9b). Moreover, XCR1^−^CD24^+^ splenic EPs showed no differences in the expression of DC-related TFs between Flt3L-injected WT and TopBP1^cKO^ mice (Supplementary Fig. 9c). These results suggest that TopBP1 may contribute to cDC development through mechanisms other than direct regulation of DC-related TF expression during cDC differentiation. For further analysis, we performed bulk RNA -seq transcriptomic analysis on CD24^+^ splenic EPs from WT and TopBP1^cKO^ mice. Consistently, there was no significant difference in the expression of DC-related major TFs in the CD24^+^ EPs between Flt3L-injected WT and TopBP1^cKO^ mice (Fig. 6a). By contrast, genes whose expression was reduced by more than 50% in TopBP1^cKO^ EPs compared with WT EPs were predominantly associated with protein binding in molecular functions and transcription regulation by RNA polymerase II in biological processes (Fig. 6b). It is well established that TopBP1 functions as a scaffold protein, indirectly binding to DNA through protein–protein interactions^59,60^. Based on this, we evaluated the expression of TF target genes curated from the TRRUST database and Ma’ayan Laboratory resources and found that the target genes regulated by PU.1 (Spi1), IRF8 and IRF4 were substantially downregulated (fold change <0.5, false discovery rate (FDR) <0.05) in TopBP1^cKO^ EPs. (Fig. 6c). Intriguingly, more than half of the IRF8 and IRF4 target genes downregulated in TopBP1^cKO^ EPs overlapped with the PU.1 target genes (Fig. 6c). Heat map analysis (fold change <0.1, FDR <0.05) revealed that the expression levels of PU.1/IRF8/IRF4 target genes were significantly reduced in TopBP1^cKO^ EPs compared with WT EPs, and most of the IRF4 and IRF8 target genes downregulated in TopBP1^cKO^ EPs overlapped with those of PU.1 (Fig. 6d). Taken together, our results suggest that TopBP1 plays a crucial role in cDC development by regulating the target gene expression of DC-related TFs, such as PU.1, IRF4 and IRF8, rather than by directly controlling the expression of the TFs themselves.

### TopBP1 directly interacts with PU.1–IRF8 heterodimeric complex to facilitate transcription of their target genes during cDC development

As TopBP1 does not regulate the expression of DC-specific TFs during DC differentiation, we hypothesized that it may instead interact with these TFs. TopBP1 interact with Miz-1^46^, which has been reported to associate with IRF8 and PU.1 to induce Nramp1 expression in a hematopoietic cell line^47^. Given these previous findings, we investigated whether TopBP1 interacts with PU.1 and IRF8 via Miz-1 during DC development. To investigate whether TopBP1 interacts with PU.1, IRF8 and/or IRF4 to induce target gene expression, leading to cDC development, we performed ChIP-seq analysis on CD24^+^ splenic EPs of FL-injected WT mice. In CD24⁺ EP cells, 42 target genes were identified through anti-TopBP1 antibody ChIP-seq analysis, a number smaller than expected, probably due to limited sample availability. Among these 42 genes, we analyzed PU.1, IRF8 and IRF4-regulated target genes using the Harmonizome database provided by the Ma’ayan Laboratory. Through the analysis, we identified a total of 17 genes, including 9 PU.1-specific target genes, 4 PU.1/IRF8-shared target genes, 2 PU.1/IRF4–shared target genes and 2 IRF4-specific target genes (Fig. 7a). H3K4me is widely recognized as an activating histone modification, and all 17 of these genes were detected in anti-H3K4me antibody ChIP-seq datasets (Fig. 7a). Subsequent qRT–PCR analysis confirmed a significant reduction in the expression of PU.1-specific target genes (*Ch25h*, *Bambi*, *Tdrd3 and Mllt3*) and PU.1/IRF4-shared target gene (*Nek1*) in the XCR1^−^CD24^+^ splenic EPs of TopBP1^cKO^ mice compared with WT mice (Fig. 7b). Biological functions of these target genes remain to be elucidated in relation to cDC1 development. Intriguingly, although the protein levels of IRF8 in both pre-DCs and splenic EPs (cDC1 and XCR1^−^CD24^+^ EPs) were even higher in TopBP1^cKO^ mice than in WT mice (Supplementary Fig. 9d), cDC1s were still deficient in TopBP1^cKO^ mice (Figs. 2d and 4c), suggesting that TopBP1 is essential for IRF8-mediated cDC1 differentiation. Next, we performed co-immunoprecipitation (Co-IP) assays and found that TopBP1 directly interacts with both PU.1 and IRF8 but shows weak interaction with IRF4 and no detectable interaction with Miz-1 in WT CD24^+^ splenic EPs from Flt3L-injected mice and in in vitro-generated FL-BMDCs (Fig. 7c). These interactions were further assessed in steady-state splenic CD11c⁺ cells and revealed normal interactions not only with PU.1 and IRF8 but also with IRF4 under steady-state conditions (Supplementary Fig. 10). Confocal microscopy further revealed strong overlapping fluorescence signals of TopBP1 with PU.1 and IRF8, whereas only weak overlap was observed with IRF4, in WT FL-injected splenic CD11c⁺ cells (Fig. 7d). In a correlation analysis with the Coloc 2 plugin in the Fiji program, TopBP1 showed a strong correlation with PU.1 (*r* = 0.84 ± 0.05) and IRF8 (*r* = 0.76 ± 0.09), whereas its colocalization with IRF4 was significantly lower (*r* = 0.11 ± 0.07) in FL-injected WT splenic CD11c⁺ cells (Fig. 7e). Taken together, these results suggest that TopBP1 does not interact with Miz-1 but instead strongly interacts with PU.1 and IRF8 and weakly interacts with IRF4 in the splenic DCs of FL-injected mice, probably reflecting the Flt3L-injected condition, which favors cDC1 differentiation and reduces IRF4⁺ cDC2 abundance. To further assess whether these interactions observed in mice are conserved in humans, we transfected HEK293 cells with human-derived TopBP1, PU.1, IRF8, IRF4 and Miz-1 expression vectors. The Co-IP assay revealed that human TopBP1 also interacts with human PU.1 and IRF8 (Fig. 7f) as well as IRF4 (Supplementary Fig. 11a) but not with Miz-1 (Supplementary Fig. 11b), suggesting a potential role for these interactions in human cDC development. PU.1 and IRF8 are known to form a heterodimeric complex that co-binds to specific target genes, thereby regulating inflammatory responses, antimicrobial immunity, anti-tumor immunity and cell differentiation^61^. In our mRNA-seq analysis, approximately 80% of IRF8 target genes were found to overlap with PU.1 target genes (Fig. 6c, d), promoting us to investigate whether TopBP1 is directly involved in the formation of the PU.1–IRF8 heterodimeric complex. However, ectopic expression experiments demonstrated that PU.1 and IRF8 can form a complex even in the absence of TopBP1 (Fig. 7g), suggesting that TopBP1 is not essential for the formation of the PU.1–IRF8 heterodimeric complex but is probably required for their function in regulating the transcription of target genes. On the other hand, IRF8 has been reported to suppress neutrophil (NP) differentiation, thereby acting as a key regulator of the developmental balance between DCs and NPs^62^. Given that cDC differentiation is severely impaired in TopBP1^cKO^ mice, we examined whether NP differentiation is concomitantly enhanced under TopBP1-depleted conditions. We first examined the expression of TopBP1 and CD11c in splenic NPs. TopBP1 was highly expressed in splenic NPs (Supplementary Fig. 12a), and approximately 35% of NPs displayed intermediate levels of CD11c expression (Supplementary Fig. 12b). We also assessed splenic NP populations in TopBP1^cKO^ mice and observed a substantial increase in NPs compared with WT mice (Supplementary Fig. 12c). These findings suggest that the expansion of the splenic NP population in CD11c-specific TopBP1^cKO^ mice may result from impaired IRF8-mediated suppression of NP differentiation due to TopBP1 depletion. Further study remains to elucidate the role of TopBP1 in IRF8-mediated NP suppression, as it lies beyond the scope of the present study.

Collectively, our findings demonstrate that interactions between TopBP1 and the PU.1–IRF8 heterodimeric complex are critical for cDC development, particularly for the differentiation of cDC1s during Flt3L-based immunotherapy.

## Discussion

TopBP1 has been reported to be highly expressed in cancer patients, leading to suggestion of TopBP1-targeting tumor therapy. In the present study, we find that TopBP1 is also highly expressed in cDCs and their precursors. To evaluate the role of TopBP1 in cDC development, we generated TopBP1^cKO^ mice exhibiting rapid tumor growth, attributed to impaired anti-tumor immunity compared with WT mice. The diminished anti-tumor immunity in TopBP1^cKO^ mice was associated with a dramatic reduction in cDC populations compared with WT mice. Notably, both cDC1 and cDC2 subsets were markedly reduced in number and population in TopBP1^cKO^ mice, whereas the pDC population remained unchanged. In addition, we found that TopBP1 is essential for the development of MHCII^+^ cells during differentiation of pre-DCs into cDCs under steady-state conditions.

Moreover, the reduced cDC population in TopBP1^cKO^ mice was not due to impaired proliferation or increased apoptosis in pre-DCs or cDCs within TopBP1-deficient DC lineages. TopBP1, a scaffold protein known for its role in DNA damage repair of DSBs^19^, showed no detectable accumulation of DSBs in pre-DCs or cDCs of TopBP1^cKO^ mice compared with WT mice. These findings suggest that the reduction of cDC population upon TopBP1 depletion in TopBP1^cKO^ mice is unlikely to be due to the mechanisms previously reported in B and T cell development^19^, where it functions by repairing DSBs during V(D)J recombination to support differentiation.

Flt3L-based tumor immunotherapy was ineffective in suppressing tumor growth in TopBP1^cKO^ TBM, primarily due to a deficiency of EP-derived cDC1s, despite the EP population being comparable between Flt3L-injected TopBP1^cKO^ and WT mice. Our results highlight TopBP1 as essential for the final differentiation process of XCR1^-^CD24^+^ EPs into XCR1^+^CD24^+^cDC1s during EP-derived cDC1 development.

To uncover the detailed mechanisms underlying TopBP1-associated cDC differentiation, we first evaluated the expression levels of several TFs well established for their role in DC differentiation in pre-DCs and cDCs of TopBP1^cKO^ mice. We did not observe any clear decrease in the expression levels of DC-associated TFs in TopBP1^cKO^ mice compared with WT mice both either steady-state or Flt3L-injected conditions. These findings were further supported by bulk RNA-seq analysis, which showed similar results. IRF8 is a well-established master regulator of cDC1 differentiation. Notably, although IRF8 expression in splenic EPs was higher in Flt3L-injected TopBP1^cKO^ mice than in WT mice, cDC1 differentiation was severely impaired. These findings suggest that TopBP1 is indispensable for IRF8-mediated cDC1 development from EPs. DAVID-based gene ontology analysis with the bulk RNA-seq data revealed a significant reduction in the expression of genes involved in protein binding and transcription regulation by RNA polymerase II in CD24^+^ EPs of TopBP1^cKO^ mice compared with WT mice. Further analysis of the bulk RNA-seq data focusing on the target genes of DC-associated TFs showed that PU.1-, IRF4- and IRF8-specific or PU.1-, IRF4- and IRF8-shared target genes were significantly downregulated in TopBP1^cKO^ mice, with PU.1 target genes being most frequently detected.

IRF8 is also well known to regulate the survival and function of both cDCs and pDCs^30^. However, unlike cDCs, the population and number of pDCs remained unchanged across several organs in TopBP1^cKO^ mice compared with WT mice (Fig. 2e). These results suggest that although TopBP1 plays a crucial role in cDC development via its interactions with IRF8 and PU.1, it may not be required for IRF8-mediated pDC development.

According to previous reports, TopBP1 is known to interact with Miz-1^46^. However, our study showed that TopBP1 does not interact with Miz-1. Miz-1 plays a role in cell cycle arrest by promoting the expression of cell cycle inhibitor proteins in response to DNA damage^63^. Under normal conditions, Miz-1 binds to TopBP1, preventing its proteasomal degradation and facilitating its chromatin association and stability, thereby repressing the transcription of cell cycle inhibitors such as p21. By contrast, under stress conditions such as UV exposure, Miz-1 dissociates from TopBP1, leading to increased p21 transcription and cell cycle arrest^46^. However, pre-DCs and cDCs are almost at the terminal differentiation stage and are largely independent of the cell cycle, which may explain why TopBP1 does not interact with Miz-1 in these cell types. Instead, our findings in ChIP-seq and Co-IP analyses suggest that TopBP1 is essential for the function of IRF8-PU.1 heterodimeric TFs.

When TopBP1 interacts with IRF8-PU.1 heterodimeric complex during DC development under steady-state conditions, the TopBP1–PU.1 axis is expected to play an essential role at an early stage of DC development, thereby leading to a substantial reduction in both cDC1 and cDC2 in TopBP1^cKO^ mice. Indeed, the mixed BM adoptive transfer experiment shown in Fig. 3, demonstrates that pre-DCs accumulate whereas cDCs are drastically reduced in TopBP1^cKO^ mice, making it difficult to discern subset-specific effects of TopBP1 on cDC1 versus cDC2. This observation is consistent with a previous report showing that both cDC1 and cDC2 cell numbers are profoundly decreased in PU.1-deficient mice^43^. By contrast, during Flt3L therapy in WT TBMs, the TopBP1–IRF8 axis plays a critical role, leading to a marked expansion of EPs (Fig. 4d) and a substantial increase in the IRF8-dependent cDC1 population (Fig. 4c). Accordingly, a significant reduction in cDC1 was clearly observed in TopBP1^cKO^ mice (Fig. 4f).

CIITA promoter I (CIITApI) is well established as a key regulator of type 1 CIITA (CIITA1) expression, which drives MHCII expression during DC development^64^. PU.1^KO^ mice have been reported to exhibit a reduction in total cDCs, including both cDC1 and cDC2 subsets, compared with WT mice, along with decreased expression of *Ciita* in PU.1-deficient cDCs^43^. In our study, despite comparable expression levels of PU.1 and IRF8 between WT and TopBP1^cKO^ mice, TopBP1^cKO^ mice exhibited a marked reduction in total cDCs (Fig. 2a), as well as impaired differentiation from MHCII⁻ to MHCII⁺ populations (Figs. 3f, 4e). These findings suggest that TopBP1 is required for the proper function of PU.1 during cDC development, potentially through direct interaction, and that loss of TopBP1 compromises PU.1-mediated transcriptional activity in vivo. Specifically, TopBP1 is essential for PU.1-driven CIITApI-mediated MHC class II expression during cDC differentiation.

Although it remains controversial whether TopBP1 can directly bind to DNA, most studies indicate that TopBP1 functions primarily as a scaffold protein, facilitating protein–protein interactions via its BRCA1 C-terminal (BRCT) domains rather than directly binding to DNA^14,65^. Through TopBP1 ChIP-seq analysis, Co-IP and confocal microscopy, we demonstrated that TopBP1 directly interacts with the PU.1–IRF8 complex to promote the expression of their target genes, which is essential for total cDC development and Flt3L-mediated cDC1 differentiation (Fig. 7a–d). The specific BRCT domains of TopBP1 responsible for mediating its interactions with PU.1 and IRF8 remain to be elucidated in future studies.

Notably, TopBP1–PU.1–IRF8 interactions observed in mice were also replicated in human cells. These findings suggest that TopBP1 plays a crucial role in cDC development, particularly in promoting cDC1-derived anti-tumor immunity in patients with cancer receiving Flt3L tumor immunotherapy. In this context, *TopBP1* SNPs could serve as potential biomarkers for predicting the efficacy of Flt3L immunotherapy.

In summary, we identified TopBP1 as essential for the differentiation of pre-DCs into cDCs and for Flt3L-mediated cDC1 development. Specifically, TopBP1 plays a crucial role in the differentiation of XCR1^−^CD24^+^cDC1 EPs into XCR1^+^CD24^+^ cDC1s in Flt3L immunotherapy. TopBP1 regulates the expression of PU.1 and IRF8 target genes through direct interaction with these TFs. These findings underscore the indispensable role of TopBP1 in cDC development, particularly in Flt3L-mediated differentiation of EPs into cDC1s. The detailed molecular mechanisms by which TopBP1 interacts with PU.1 and IRF8 remain to be elucidated.

## Supplementary information

Supplementary Information
