# Supplementary material for: TopBP1 orchestrates PU.1–IRF8 transcriptional programming of dendritic cell differentiation and Flt3L-driven tumor immunity
Source: Exp Mol Med. 2026 May 8;58(5):1556–72. doi: 10.1038/s12276-026-01715-1 (PMC13234112; doi:10.1038/s12276-026-01715-1)
Supplement: Supplementary file 1 — Supplementary Information [file 12276_2026_1715_MOESM1_ESM.pdf]

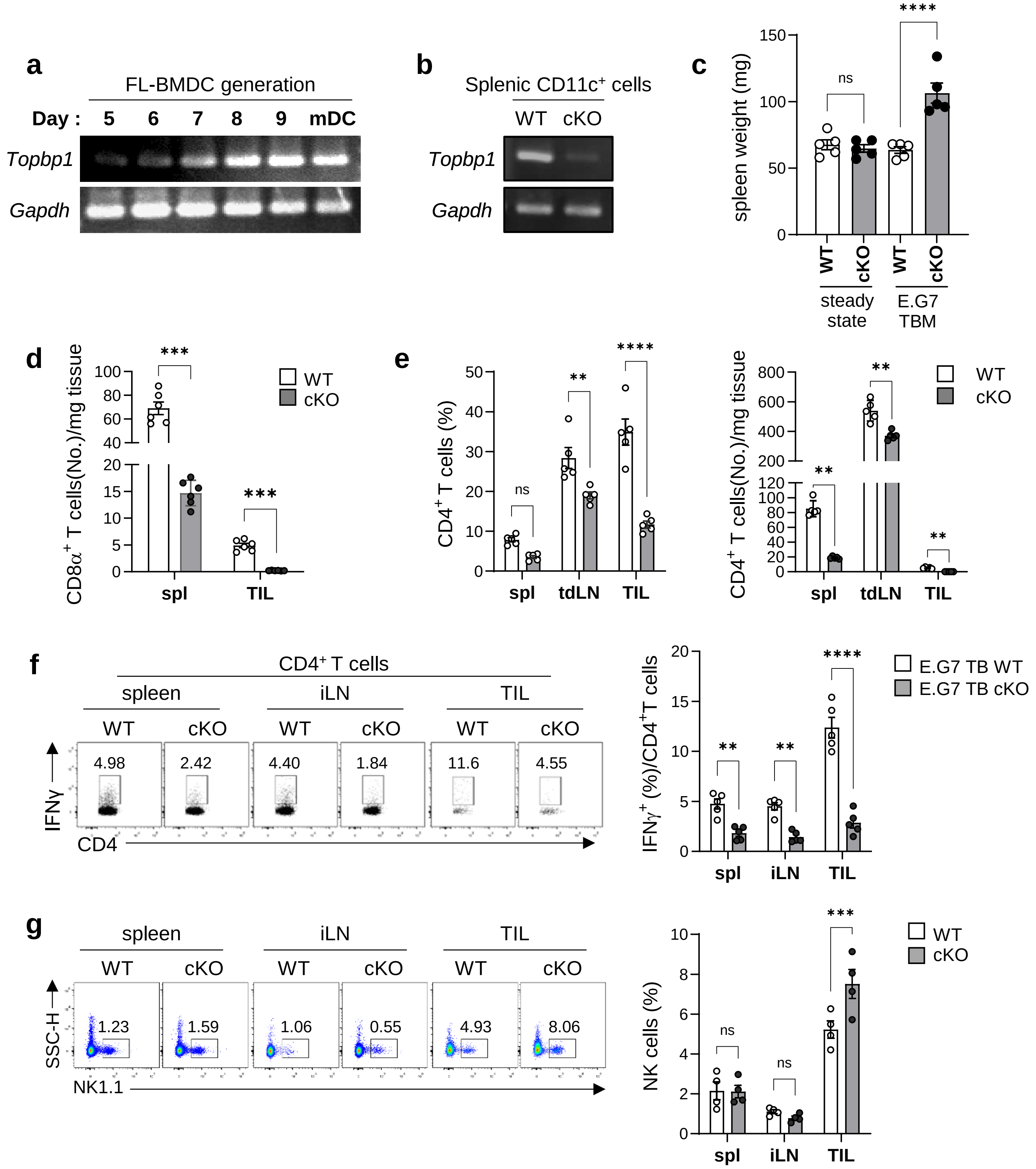

Supplementary Fig. 1. Analysis of immune cells in WT and TopBP1^{cKO} E.G7 TB mice.

a RT-PCR analysis of TopBP1 expression during Flt3L(50ng/mL)-BMDC generation. **b** TopBP1 expression in the splenic CD11c-positive cells sorted from WT and TopBP1^{cKO} mice with magnetic beads. **c** Weight of the spleen in WT and TopBP1^{cKO} mice in steady state and tumor-bearing conditions. **d** CD8α^{+} T cells in the organs of WT and TopBP1^{cKO} E.G7 TB mice. *n* = 5 per group. **e** CD4^{+} T cells in the organs of WT and TopBP1^{cKO} E.G7 TB mice. *n* = 5 per group. **f** IFNγ^{+} CD4^{+} T cells in the organs of WT and TopBP1^{cKO} E.G7 TB mice. *n* = 5 per group. **g** NK cells in the organs of WT and TopBP1^{cKO} E.G7 TB mice. *n = 4* per group. Two-way ANOVA with Tukey's multiple comparisons test (c-g) was used to measure significance. $^{**}P$ <0.01, $^{***}P$ <0.001, $^{****}P$ <0.0001 ns; not significant, error bars indicate mean ± SEM.

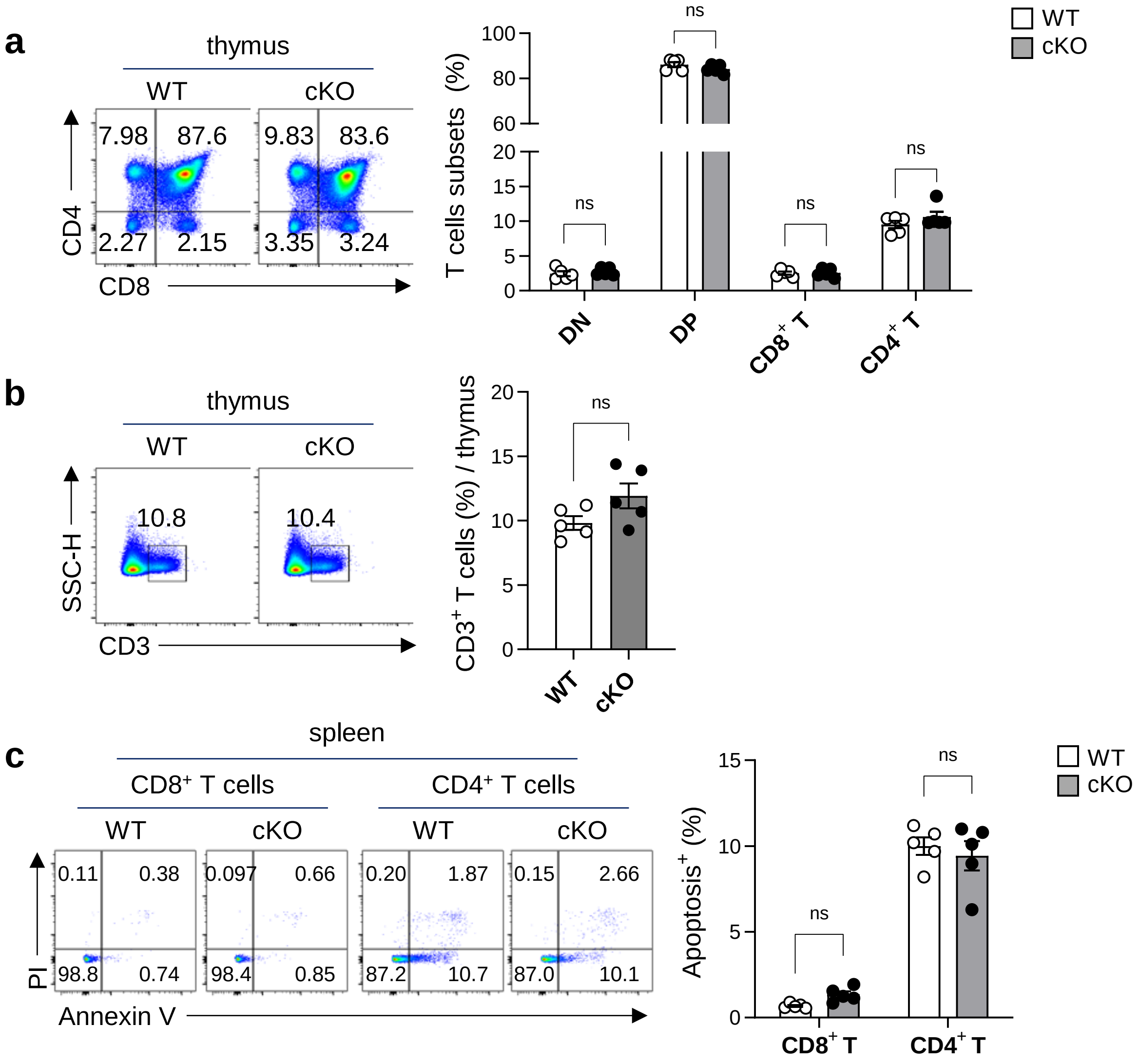

Supplementary Fig. 2. T cell development and apoptosis in WT and TopBP1^{cKO} mice.

a T cells development analysis in thymus of WT and TopBP1^{cKO} mice. *n* = 5 per group. **b** CD3^{+} T cells in thymus of WT and TopBP1^{cKO} mice. *n* = 5 per group. **c** Cell apoptosis in T cells subsets from the spleens of WT and TopBP1^{cKO} mice. *n* = 5 per group. Unpaired t-test with Welch's correction (b) and Two-way ANOVA with Bonferroni (a) or Tukey's multiple comparisons test (c) were used for statistics. ns; not significant, error bars indicate mean ± SEM.

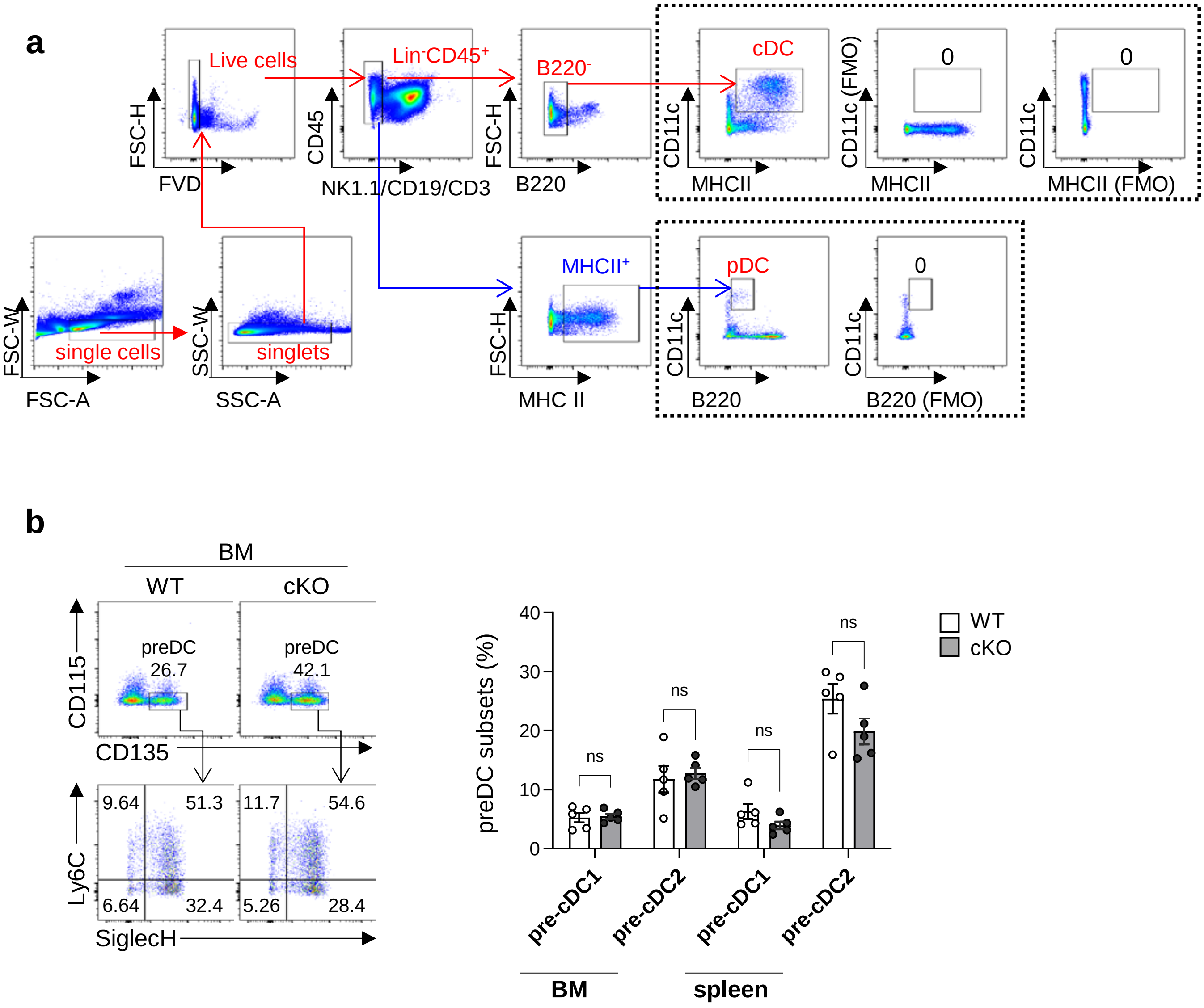

Supplementary Fig. 3. Gating strategy for DC subset and analysis of pre-DCs subsets in the organs.

a Gating strategy for DC subsets at steady state. Doublet cells were excluded using FSC-W vs FSC-A, and SSC-W vs SSC-A. Dead cells were excluded using FVD vs FSC-H. **b** Pre-DC subsets were assessed in the BM and spleens of WT and TopBP1^{cKO} mice. *n* = 4 per group. Two-way ANOVA with Turkey's multiple comparisons test was used to measure significance. ns; not significant, error bars indicate mean ± SEM.

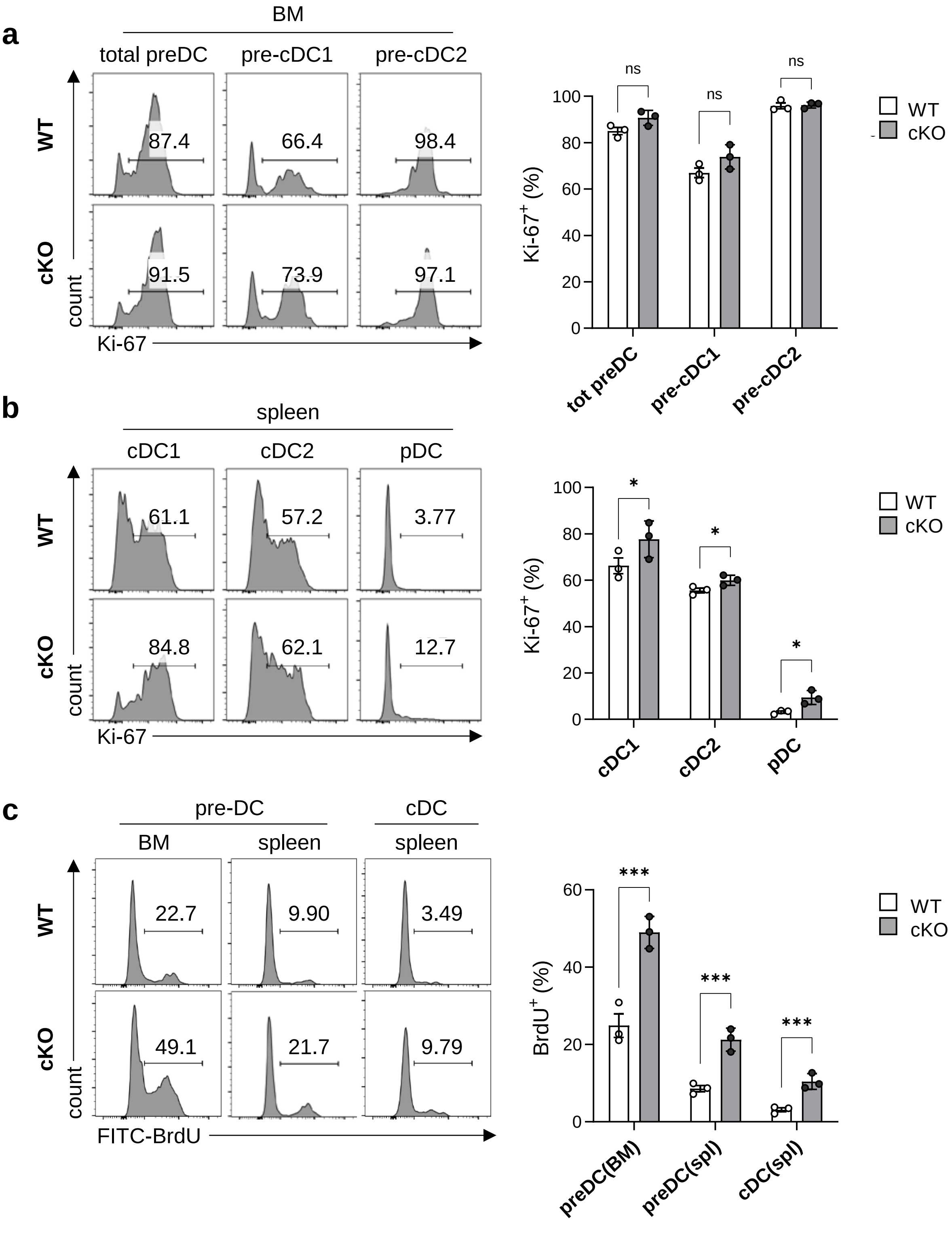

Supplementary Fig. 4. Analysis of proliferation

a Percent of Ki-67⁺ population in total pre-DC, pre-cDC1, and pre-cDC2 in BM of WT and TopBP1^{cKO} mice. **b** Percent of Ki-67⁺ population in splenic cDC1, cDC2, and pDC of WT and TopBP1^{cKO} mice. **c** Results of a BrdU incorporation assay in pre-DCs and cDCs from the BM and spleens of WT and TopBP1^{cKO} mice. Two-way ANOVA with Tukey's multiple comparisons test (a-c) was used for statistics. n = 3 per group. *P <0.05, ***P <0.001, ns; not significant, error bars indicate mean ± SEM.

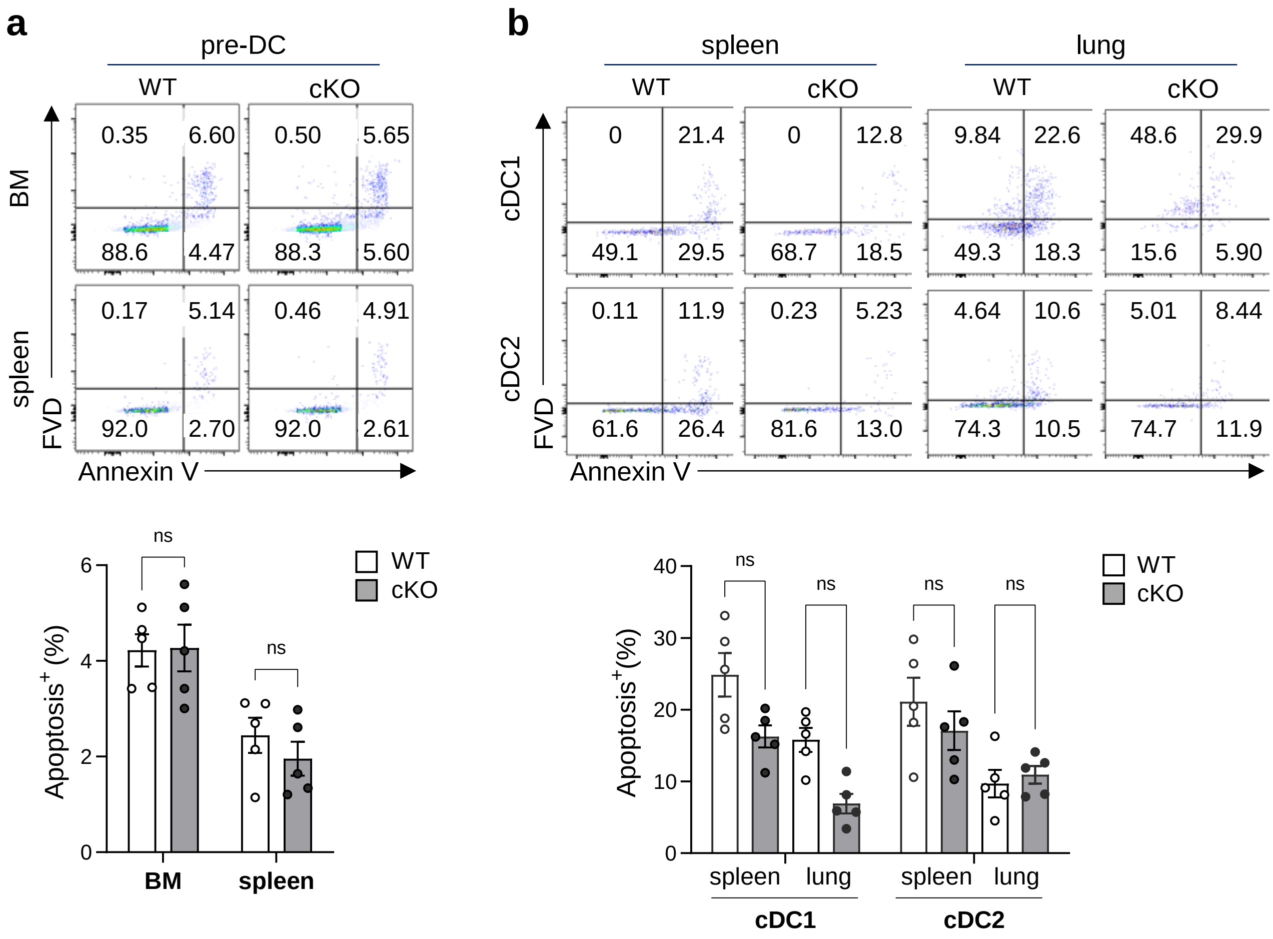

Supplementary Fig. 5. Analysis of apoptosis.

a Cell apoptosis in pre-DCs from the BM and spleens of WT and TopBP1^{cKO} mice. *n* = 5 per group. **b** Cell apoptosis in cDC subsets isolated from the spleens and lungs of WT and TopBP1^{cKO} mice. *n* = 5 per group. Two-way ANOVA with Tukey's multiple comparisons test (a, b) were used for statistics. ns; not significant, error bars indicate mean ± SEM.

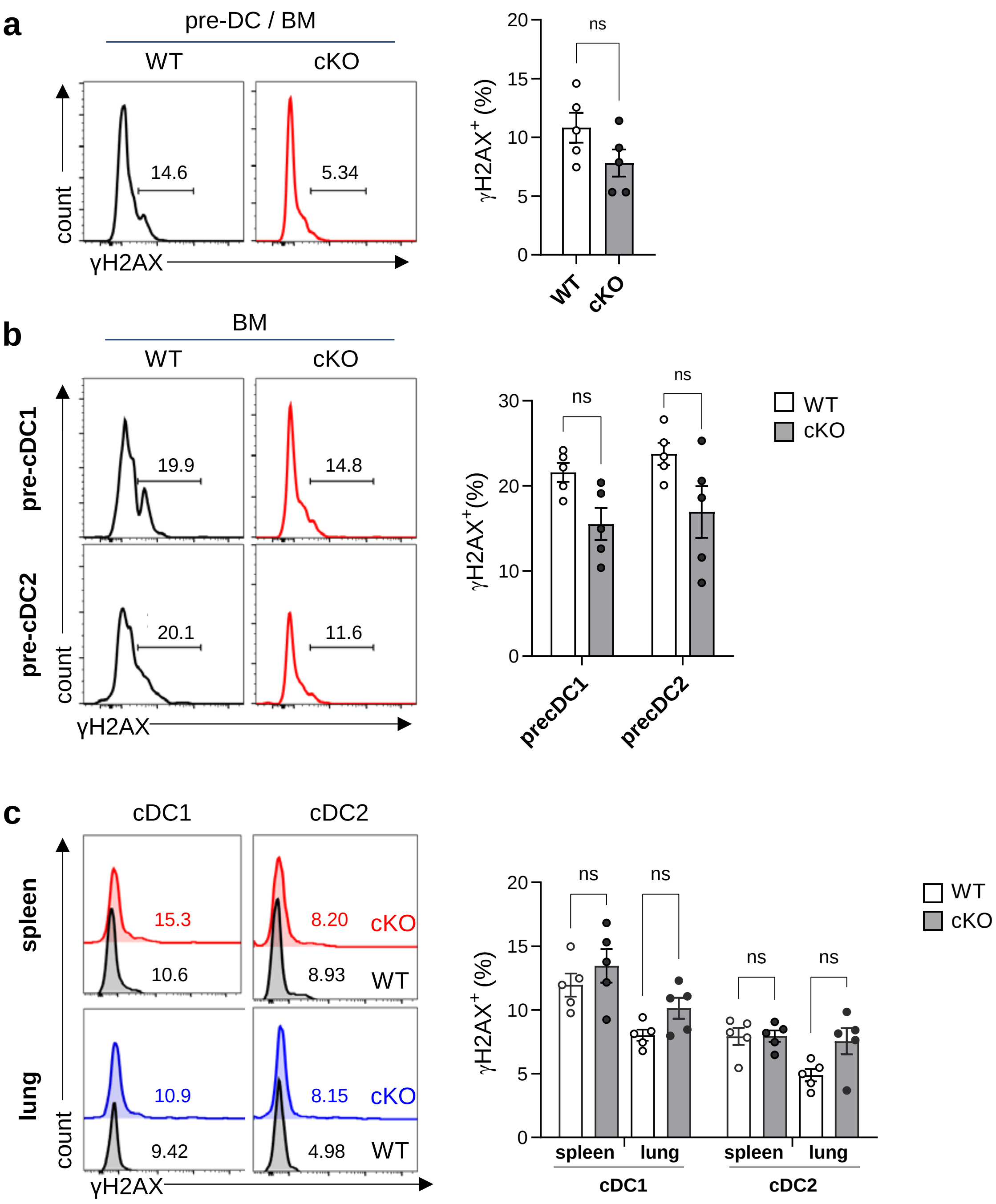

Supplementary Fig. 6. Analysis of DNA damage.

a DNA damage (γH2AX⁺) analysis in BM pre-DCs of WT and TopBP1^{cKO} mice. *n* = 5 per group. **b** DNA damage (γH2AX⁺) analysis in BM pre-DC subsets of WT and TopBP1^{cKO} mice. *n* = 5 per group. **c** DNA damage (γH2AX⁺) analysis in cDC1s, cDC2s, and pDCs in the spleens and lungs of WT and TopBP1^{cKO} mice. *n* = 5 per group. Unpaired t-test with Mann-Whitney test (a) and Two-way ANOVA with Tukey's multiple comparisons test (b, c) were used for statistics. ns; not significant, error bars indicate mean ± SEM.

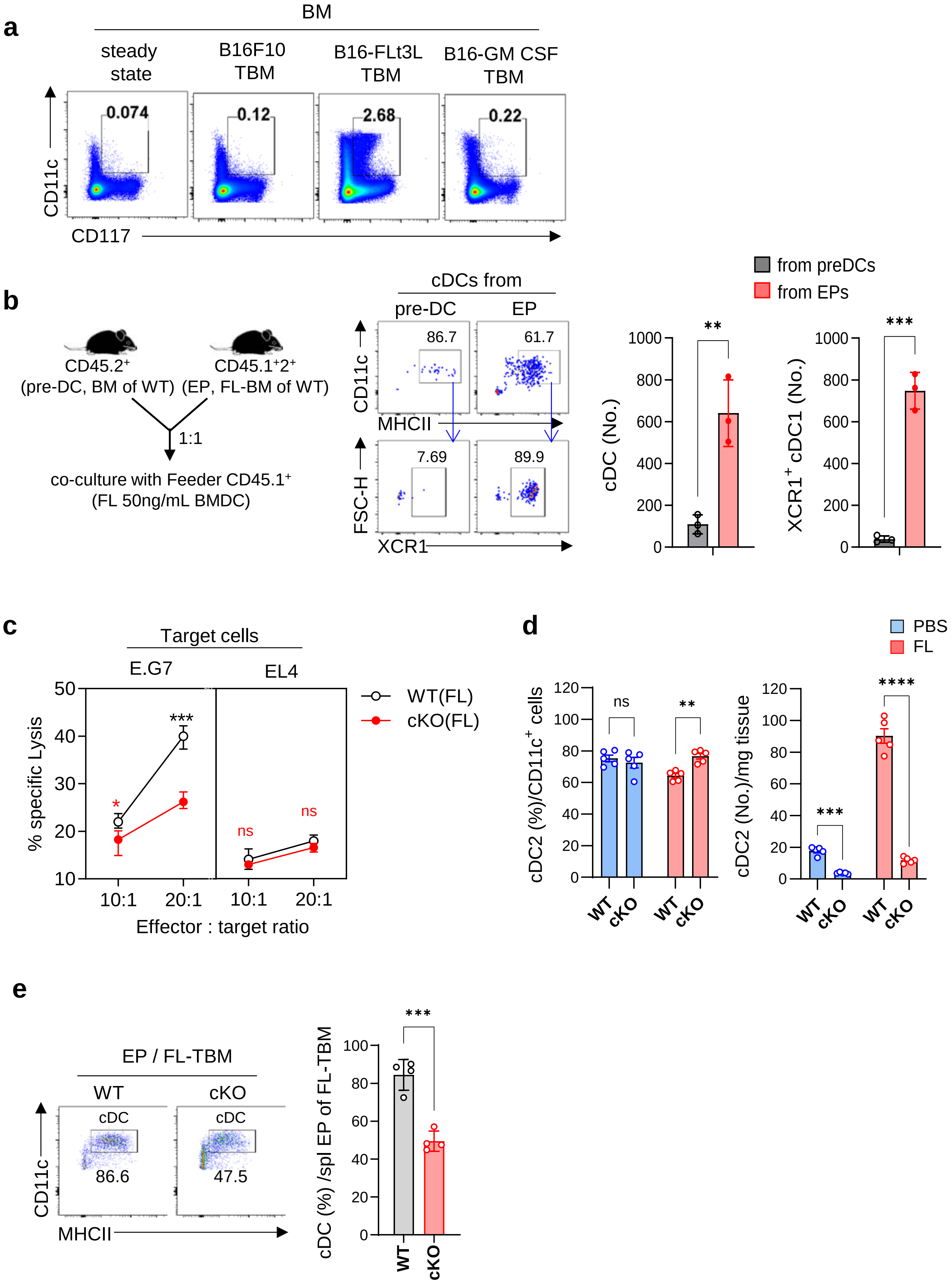
a
BM
steady state
B16F10 TBM
B16-FLt3L TBM
B16-GM CSF TBM
0.074
0.12
2.68
0.22
CD11c
CD117
b
CD45.2+
(pre-DC, BM of WT)
CD45.1+2+
(EP, FL-BM of WT)
1:1
co-culture with Feeder CD45.1+
(FL 50ng/mL BMDC)
cDCs from
pre-DC
EP
86.7
61.7
7.69
89.9
CD11c
MHCII
FSC-H
XCR1
from preDCs
from EPs
cDC (No.)
XCR1+ cDC1 (No.)
c
Target cells
E.G7
EL4
% specific Lysis
WT(FL)
cKO(FL)
Effector : target ratio
d
PBS
FL
cDC2 (%)/CD11c+ cells
cDC2 (No.)/mg tissue
WT
cKO
e
EP / FL-TBM
WT
cKO
cDC
86.6
47.5
CD11c
MHCII
cDC (%) /spl EP of FL-TBM

Supplementary Fig. 7. Flt3L-mediated of EP differentiation into cDC1s.

a EP populations in B16F10, B16F10-Flt3L, and B16F10-GM-CSF TB mice. **b** BM pre-DCs from WT ($CD45.2^+$) and BM EPs from Flt3L-injected mice ($CD45.1^+2^+$) were mixed at a 1:1 ratio and cultured with feeder cells (FL-BMDCs, day 5). After 4 days, cDCs and cDC subsets differentiated from donor cells were assessed. *n* = 3 per group. **c** CTL activity in the TILs of WT and $TopBP1^{cKO}$ E.G7 TBM treated with Flt3L. *n* = 4 per group. **d** Analysis of cDC2 frequency and number in the spleens of WT and $TopBP1^{cKO}$ E.G7 TBM treated with Flt3L. *n* = 5 per group. **e** cDC population in the splenic EPs of FL-injected WT and $TopBP1^{cKO}$ E.G7 TBM, *n* = 4 per group. Unpaired t-test with Welch's correction (b, e) and Two-way ANOVA with Tukey's multiple comparisons test (c,d) were used to measure significance. ***P* <0.01, ****P* <0.001, *****P* <0.0001, ns; not significant, error bars indicate mean ± SEM

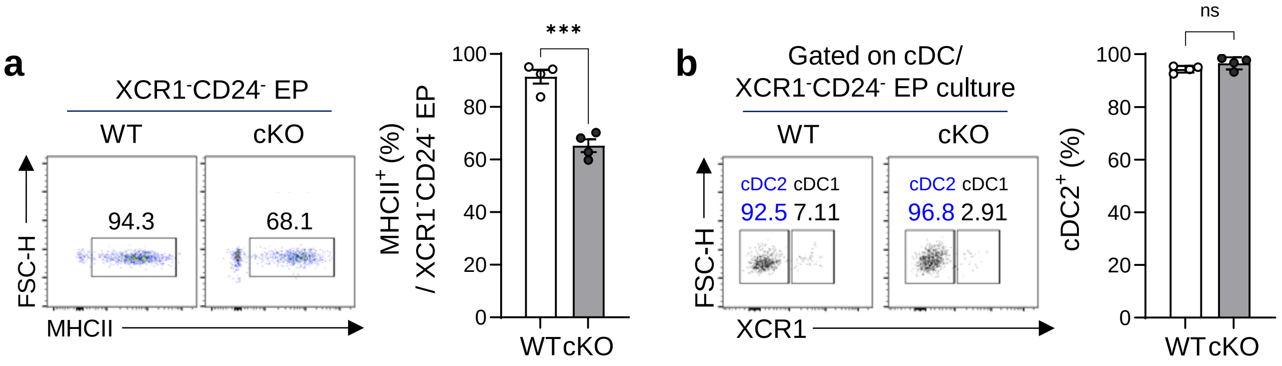

Supplementary Fig. 8. Flt3L-mediated differentiation of XCR1⁻CD24⁻ EP into cDC2s.

a XCR1⁻CD24⁻ MHCII⁺ populations in the splenic EPs of Flt3L-injected WT and TopBP1^{cKO} mice.

b XCR1⁻CD24⁻ cells sorted from splenic EPs of Flt3L-injected WT (CD45.1⁺2⁺) and TopBP1^{cKO} (CD45.2⁺) mice were mixed at a 1:1 ratio and culture with feeder cells (FL-BMDCs, day 5). After 5 days, XCR1 expression was examined in donor-derived cells. *n* = 4 per group.

Unpaired t-test with Welch's correction (a, b) was used to measure significance. ****P* <0.001, ns, not significant; error bars indicate mean ± SEM

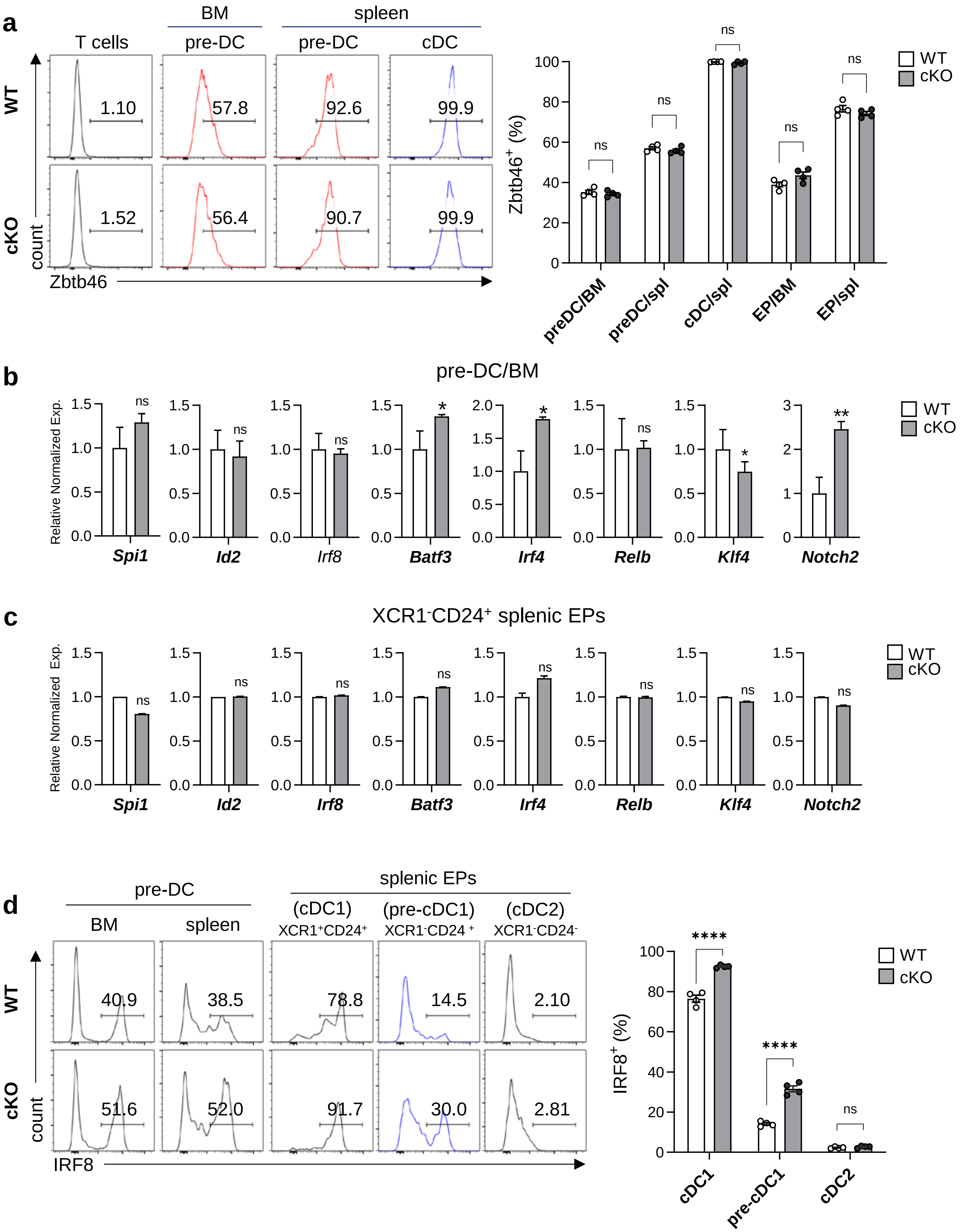
a
BM
spleen
T cells
pre-DC
pre-DC
cDC
WT
cKO
count
Zbtb46
1.10
57.8
92.6
99.9
1.52
56.4
90.7
99.9
Zbtb46+ (%)
ns
WT
cKO
preDC/BM
preDC/spl
cDC/spl
EP/BM
EP/spl
b
pre-DC/BM
Relative Normalized Exp.
Spi1
Id2
Irf8
Batf3
Irf4
Relb
Klf4
Notch2
ns
*
**
c
XCR1-CD24+ splenic EPs
Relative Normalized Exp.
Spi1
Id2
Irf8
Batf3
Irf4
Relb
Klf4
Notch2
d
pre-DC
BM
spleen
splenic EPs
(cDC1)
XCR1+CD24+
(pre-cDC1)
XCR1-CD24+
(cDC2)
XCR1-CD24-
WT
cKO
count
IRF8
40.9
38.5
78.8
14.5
2.10
51.6
52.0
91.7
30.0
2.81
IRF8+ (%)
****
ns
cDC1
pre-cDC1
cDC2

Supplementary Fig. 9. Expression of transcription factors in BM pre-DCs and splenic EPs of WT and TopBP1^{cKO} mice.

a Zbtb46 levels assessed by FACS in pre-DCs and cDCs of WT and TopBP1^{cKO} mice. **b** Relative expression levels of transcription factors, essential for cDC development, in BM pre-DCs of WT and TopBP1^{cKO} mice, assessed by RT-PCR. **c** Relative expression levels of transcription factors essential for cDC development within XCR1^{-}CD24^{+} splenic EPs of WT and TopBP1^{cKO} mice, assessed by RT-PCR. **d** IRF8 protein levels were assessed by FACS in pre-DCs and splenic EPs of WT and TopBP1^{cKO} mice. Two-way ANOVA with Tukey's multiple comparisons test (a, d) and unpaired t-test (b, c) were used to measure significance. *n* = 4 per group. *P <0.05, **P <0.01, **** P <0.0001 ns; not significant; error bars indicate mean ± SEM.

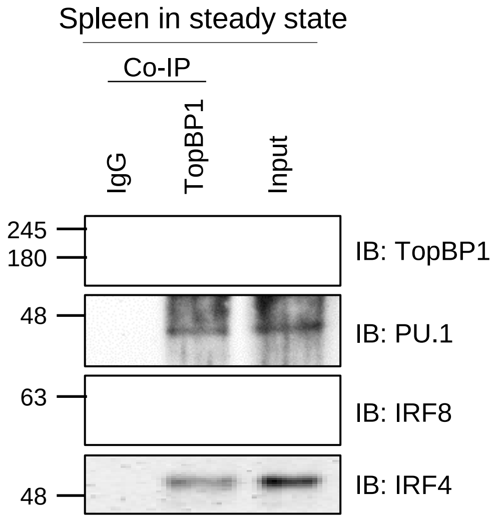

Supplementary Fig. 10. TopBP1 interacts with PU.1, IRF8, and IRF4 at steady state. Co-IP of PU.1, IRF8, and IRF4 using an anti-TopBP1 antibody in steady-state CD11c^{+} splenic cells from WT mice, followed by IB with the indicated antibodies.

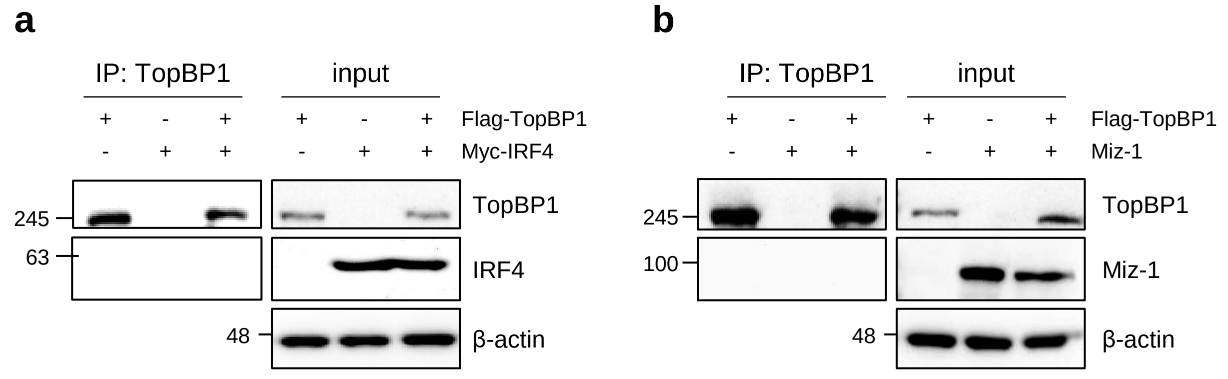

Supplementary Fig. 11. TopBP1 interacts with human IRF4 but not with Miz-1. HEK293 cells were transfected with plasmids expressing human TopBP1 alone, IRF4 or Miz-1 alone, or co-transfected with TopBP1 and either IRF4 (**a**) or Miz-1 (**b**). Co-IP was performed using an anti-TopBP1 antibody, followed by IB with the indicated antibodies.

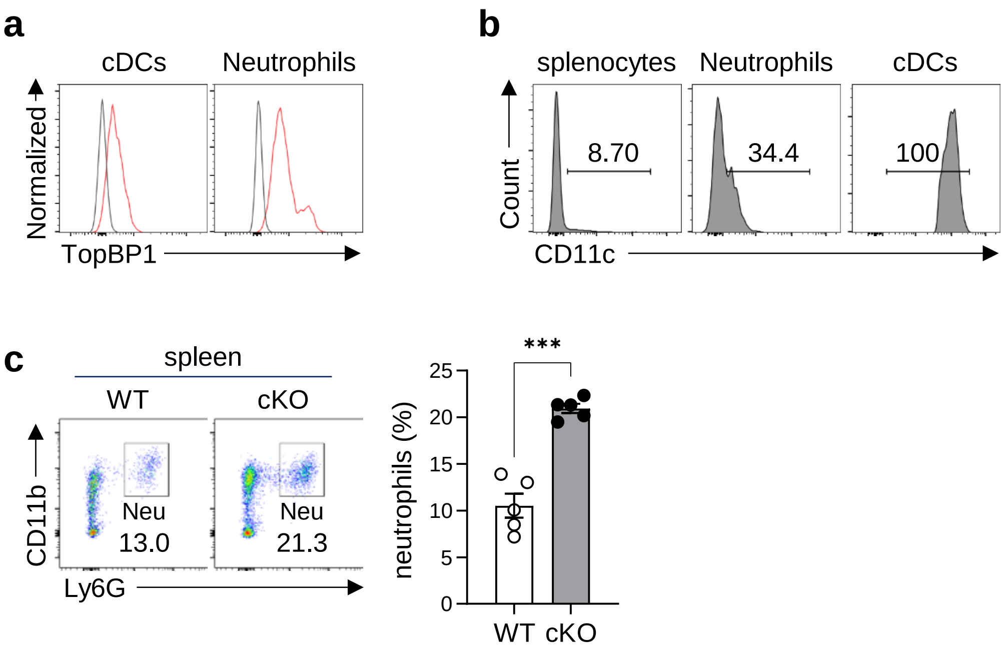

Supplementary Fig. 12. The population of splenic neutrophil increased in TopBP1^{cKO} mice. a. Analysis of TopBP1 expression in splenic neutrophils. **b** CD11c expression in splenic neutrophils. **c** the population of splenic neutrophils in WT and TopBP1^{cKO} mice. *n* = 5 per group. Unpaired t-test with Welch's correction (c) was used for statistics. ***P <0.001, error bars indicate mean ± SEM.
